# Synthesis of 1-(*para*-methoxyphenyl)tetrazolyl-Substituted 1,2,3,4-Tetrahydroisoquinolines and Their Transformations Involving Activated Alkynes

**DOI:** 10.3390/molecules23113010

**Published:** 2018-11-17

**Authors:** Alexander A. Titov, Reza Samavati, Elena V. Alexandrova, Tatiana N. Borisova, Tuyet Anh Dang Thi, Van Tuyen Nguyen, Tuan Anh Le, Alexey V. Varlamov, Erik V. Van der Eycken, Leonid G. Voskressensky

**Affiliations:** 1 Organic Chemistry Department, Peoples’ Friendship University of Russia (RUDN University), 6 Miklukho-Maklaya St., 117198 Moscow, Russia; titov_aa@pfur.ru (A.A.T.); reza_nasa3@yahoo.com (R.S.); elena-aleksandrova-00-lena@mail.ru (E.V.A.); borisova_tn@pfur.ru (T.N.B.); avarlamov@sci.pfu.edu.ru (A.V.V.); erik.vandereycken@kuleuven.be (E.V.V.d.E.); 2 Institute of Chemistry of Vietnam Academy of Science and Technology, 18 Hoang Quoc Viet, Cau Giay, Hanoi 100000, Vietnam; dangtuyetanh1201@gmail.com (T.A.D.T.); ngvtuyen@hotmail.com (V.T.N.); 3 Graduate University of Science and Technology, Vietnam Academy of Science and Technology, 18 Hoang Quoc Viet, Cau Giay, Hanoi 100000, Vietnam; 4 Faculty of Chemistry of VNU University of Science, 19 Le Thanh Tong, Hoan Kiem, Hanoi 100000, Vietnam; huschemical.lab@gmail.com; 5 Laboratory for Organic & Microwave-Assisted Chemistry (LOMAC), Department of Chemistry, KU Leuven Celestijnenlaan 200F, 3001 Leuven, Belgium

**Keywords:** tetrazolylisoquinolines, tetrazolyl benzazocines, cycle expansion, Stevens rearrangement, activated alkynes

## Abstract

1-(*p*-Methoxyphenyl)tetrazolyl-substituted 6,7-dimethoxy(6,7-methylenedioxy)-1,2,3,4-tetrahydroisoquinolines formed tetrazolyl-substituted azocines in high yields by using activated alkynes. Unsubstituted at 6,7,8-aromatic fragment 1-tetrazolylisoquinoline interacted in several pathways forming tetrazolyl-substituted azocines, 1-tetrazolyl-1-R-vinylisoquinolines and 3-azaspiro[5.5]undeca-1,7,9-triene.

## 1. Introduction

Multicomponent processes are an accessible tool for creating molecular diversity, have a high synthetic potential, and show high atom and step economy. There are many examples of isonitriles in multicomponent reactions for forming original biologically-active compounds [1,2]. They are widely used in green chemistry [3,4,5] and in the synthesis of tetrazoles [6,7,8,9,10,11]. Substituted or heterocyclic annulated tetrazoles have an extensive range of biological activity. They have exhibited anti-inflammatory [12], analgesic [12], and anticancer [13] activities. Substituted benzazocines and azocinoindoles can inhibit acetyl- and butyrylcholinesterases [14,15], and they are promising compounds for creating antineurodegenerative drugs. The combination of two pharmacophore fragments of azocine and tetrazole in one molecule can enhance previously identified, or create new activities.

## 2. Results 

Recently, we reported examples of cotarnine chloride and 2-methyl-3,4-dihydro-β-carbolinium iodide that iminium salts, can be used in multicomponent Ugi reactions [16,17]. At the 1-position of the methyl, phenyl, or benzyl substituent in the iminium salts of isoquinolines, the Ugi azido reaction is not realized. Herein, we propose to determine whether substituents in the aromatic fragment of 1-tetrazolyl-substituted isoquinolines affect the direction of transformations involving activated alkynes. Specifically, we propose the synthesis of 1-(*p*-methoxyphenyltetrazolyl)-1,2,3,4-tetrahydroisoquinoline, and its 6,7-dimethoxy- and 6,7-(methylenedioxy)-8-methoxy derivatives **1a**–**c** [16].

**Scheme 1:**
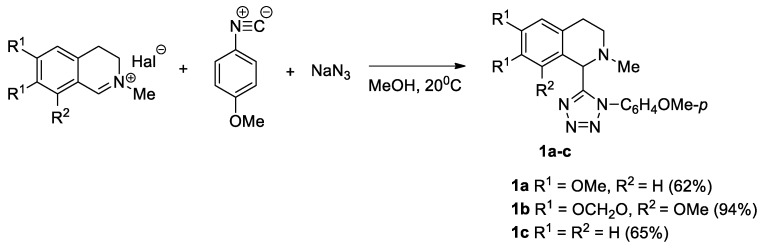
Synthesis of tetrazolyl-substituted isoquinolines **1a**–**c** via the Ugi azido reaction.

We prepared tetrazolyl-substituted isoquinolines, by reacting the corresponding iminium salt with *p*-methoxyphenyl isonitrile and sodium azide (Scheme 1).

**Scheme 2:**
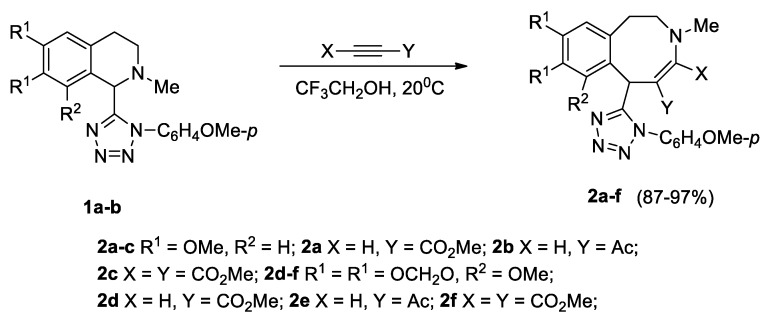
Synthesis of tetrazolyl-substituted benzazocines **2a**–**f**.

The tetrazolyl-substituted isoquinolines **1a**, **1b**, with oxygene-containing substituents in the aromatic moiety, reacted with methyl propiolate, acetylacetylene, and DMAD (dimethyl acetylenedicarboxylate) in trifluoroethanol at 20 °C for 1–12 days, affording tetrazolyl-substituted azocines **2a**–**f** in nearly quantitative yields (Scheme 2).

Previously, we obtained 6-(1-benzyl and 1-(2-ethyl-6-methylphenyl) tetrazolyl-substituted azocines from 1-tetrazolyl-2-methyl-6,7-methylenedioxy-8-methoxy-1,2,3,4-tetrahydroisoquinoline with activated alkynes, in yields of 64–75%, and with DMAD, in a yield of 94% [16].

**Scheme 3:**
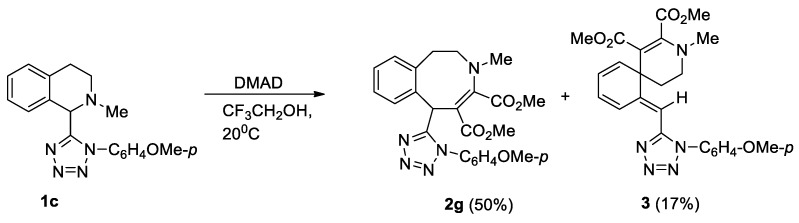
Interaction of isoquinoline **1c** with DMAD.

**Scheme 4:**
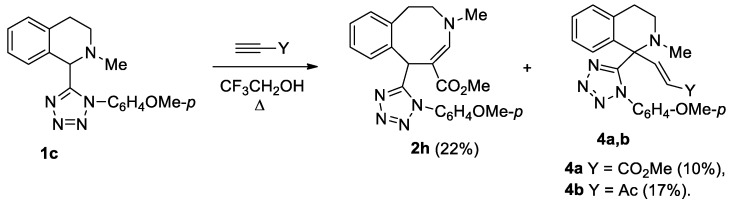
Interaction of isoquinoline **1c** with activated terminal alkynes.

Unsubstituted at the aromatic fragment, tetrahydroisoquinoline **1c** reacted difficultly and ambiguously with activated alkynes. The reaction with DMAD occurred at 20 °C over the course of 2 days (Scheme 3). In contrast, the reaction with methyl propiolate and acetylacetylene required heating to the boiling point of the solvent (Scheme 4).

Scheme 3 and Scheme 4 show transformations of isoquinoline **1c** with activated alkynes. Tetrazolyl-substituted benzazocines **2g**, **2h** formed in the reactions of compound **1c** with methyl propiolate and DMAD. We synthesized the Stevens rearrangement’s products **4a**, **4b**, using methyl propiolate and acetylacetylene, via ylides as intermediates. It was unusual to obtain spiro compound **3** from the interaction with DMAD. Previously, we reported that such spiro compounds form in the reaction of activated alkynes with tetrahydropyridines [*c*]-connected with a five-membered heterocycle, with one heteroatom condensed with a benzo- or hetaryl-containing fragment [18,19].

**Scheme 5:**
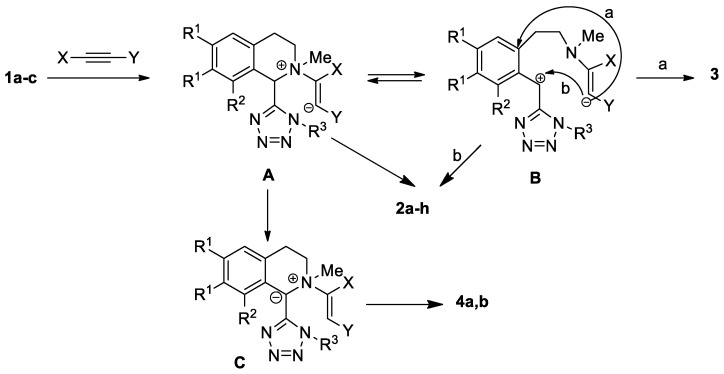
Proposed mechanism for the formation of the isolated products **2**–**4**.

We assume that the conversion of tetrazolyl-substituted isoquinolines proceeds through zwitterion **A**, which is in equilibrium with the open form **B** (Scheme 5). An interaction of ions in the intermediate **B** leads to the formation of compounds **2a**–**h**. The same products can be obtained as a result of nucleophilic attack of the cationic center in zwitterion **A** on position 1 of isoquinoline. In the reactions of methyl propiolate and acetylacetylene, the anionic center in ion **A** has a greater basicity than in the reaction of DMAD, and it can remove a proton from the C-1 position, forming ylide **C**, the rearrangement of which leads to the synthesis of vinyl-substituted tetrazolylisoquinolines **4a**, **4b**.

The formation of the unusual transformation product with DMAD (spiro compound **3**) is probably associated with a more efficient delocalization of the anionic center in intermediate **B**, which enables ipso-attack on the aromatic fragment.

**Figure 1:**
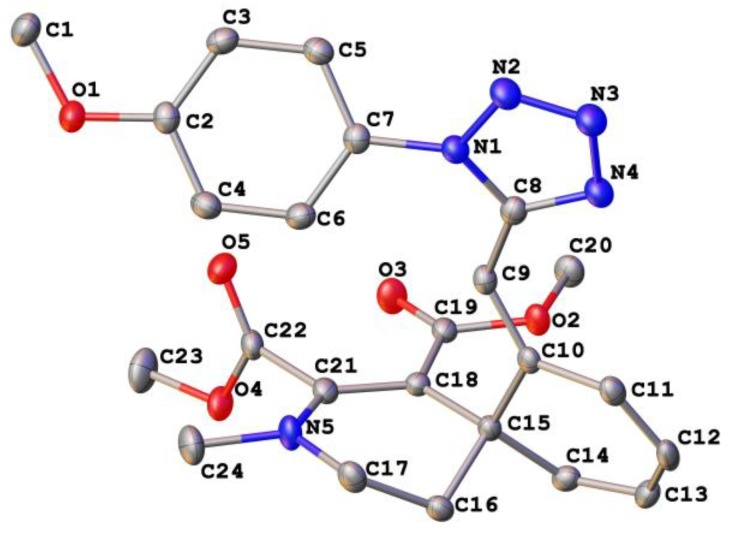
X-ray crystal diffraction of compound **3**.

We confirmed the structures of all synthesized compounds by spectral data. We characterized the ^1^H-NMR spectra of all tetrazolyl-substituted azocines by the presence of the weakfield singlets of H-6 protons at δ 6.30–6.01 ppm, and the spectra of azocines **2a**, **2b**, **2d**, **2e**, **2g** by the presence of singlets at δ 7.45–7.26 ppm, due to the presence of enamine proton H-4. The ^1^H-NMR spectra of isoquinolines **4a**, **4b** exhibits two doublets, δ 7.83–7.73 ppm and δ 5.54–5.35 ppm, with *J* = 16.2 Hz, indicating transposition of protons at a double bond (See the Supplementary Materials for further details). We confirmed the structure of spiro-compound **3** by X-ray analysis (Figure 1).

The tetrahydropyridine ring of the spiro compound **3** assumes a slightly distorted “sofa” conformation, with the carbon atom C(16) extending from the plane formed by the remaining atoms of the ring by 0.658 Å. Carbonyl fragment C(19)–O(3) of the ester group is essentially coplanar with the basal plane of the tetrahydropyridine ring C(15)–C(18)=C(21)–N(5)–C(17) (the dihedral angle is equal to 6.40°), due to bond conjugation. The nitrogen atom N(5) has a trigonal planar configuration (the sum of the valence angles is 359.7°). The cyclohexadiene ring of the spiro compound **3** assumes a slightly distorted “sofa” conformation, with the carbon atom C(15) extending from the formed by the remaining atoms of the ring by 0.444 Å. The 4-methoxyphenyl substituent in molecule **3** is twisted with a tetrazole ring at an angle of 34.20°. The molecule of spiro compound **3** contains the asymmetric carbon atom C(15). The molecules of compound **3** in the crystal form centrosymmetric dimers, due to two intermolecular hydrogen bonds C(5)–H(5)···O(5)*. 

To conclude, oxygen-containing substituents in the aromatic fragment at the tetrazolyl-substituted tetrahydroisoquinolines promote the formation of tetrazolylbenzazocines. Unsubstituted isoquinoline interacts in a more complex manner, and via several pathways.

## 3. Experimental Section 

IR spectra were registered on a Fourier spectrometer Infralum FT-801 in KBr pellets (ISP SB RAS, Novosibirsk, Russia). ^1^H and ^13^C-NMR spectra were acquired on a JEOL JNM–ECA 600 spectrometer (JEOL Ltd., Tokyo, Japan) (with operating frequencies of 600 and 150 MHz, respectively) in CDCl_3_ and DMSO-*d*_6_ solution at 23 °C. Signal of the residual protons of the solvent (7.26 ppm for CHCl_3_) was used as the reference in ^1^H-NMR spectra, while solvents signals (77.2 ppm for CDCl_3_, 39.4 ppm for DMSO-*d*_6_) were used as the reference in ^13^C-NMR spectra. Mass spectra were recorded with LCMS-8040 Triple quadrupole liquid chromatograph mass-spectrometer from Shimadzu (Shimadzu Corporation, Tokyo, Japan). Elemental analysis was performed on a EuroVector EA-3000 elemental Analyzer (Eurovector, S.p.A., Milan, Italy). Melting points were determined by the open capillary method on a Stuart SMP10 apparatus (Bibby Sterilin Ltd., Stone, UK). X-ray diffraction data were obtained on an automatic three-circle Bruker APEX-II CCD diffractometer (Bruker AXS GmbH, Karlsruhe, Germany). Sorbfil PTX-AF-A-UV plates (Imid Ltd., Krasnodar, Russia) were used for thin-layer chromatography and visualization with the iodine vapor. Column chromatography was performed on silica gel 40–60 µm, 60 Å. DMAD, methyl propiolate, acetylacetylene (Acros Organics, Geel, Belgium) and trifluoroethanol (SIA “P&M-Invest” Ltd., Moscow, Russia) were used without further purification. All solvents were distilled before use.

### 3.1. Synthesis of 1-Tetrazolyl-Substituted Isoquinolines ***1a**–**c*** (General Method) 

Sodium azide (0.72 g, 11 mmol) was added to a solution of cotarnine chloride (2.12 g, 8.3 mmol) or 6,7-dimethoxy-2-methyl-3,4-dihydroisoquinolinium iodide (2.77 g, 8.3 mmol) or 2-methyl-3,4-dihydroisoquinolinium iodide (2.27 g, 8.3 mmol) and *p*-methoxyphenyl isonitrile (1.49 g, 11 mmol) in methanol (25 mL) at 20 °C. Stirring was continued at room temperature, monitoring of the reaction progress was performed by TLC (EtOAc-hexane, 1:2). Cotarnine chloride and dimethoxy isoquinolinium iodide react for 1 day; unsubstituted aromatic fragment isoquinolinium iodide reacts for 2 days. The methanol was removed in vacuo, the residue was crystallized from a mixture of EtOAc-hexane (1:2) to afford 1-tetrazolyl-substituted isoquinolines **1a**–**c**.

*6,7-Dimethoxy-1-[1-(4-methoxyphenyl)-1H-tetrazol-5-yl]-2-methyl-1,2,3,4-tetrahydroisoquinoline* (**1a**), Yield 1.96 g (62%); beige solid; m.p. = 108–110 °С; *R*_f_ = 0.48 (EtOAc-hexane, 1:1); ^1^H-NMR (600 MHz, CDCl_3_): δ 6.95 (d, *J* = 7.8 Hz, 2H, H-Ar), 6.75 (d, *J* = 7.8 Hz, 2H, H-Ar), 6.42 (s, 1H, H-8), 6.15 (s, 1H, H-5), 5.05 (s, 1H, H-1), 3.79 (s, 6H, OCH_3_), 3.64 (s, 3H, OCH_3_), 2.94–2.91 (m, 1H, 3-CH_2_), 2.53–2.49 (m, 1H, 3-CH_2_), 2.46–2.43 (m, 2H, 4-CH_2_), 2.25 (s, 3H, N–CH_3_); ^13^C-NMR (150 MHz, CDCl_3_): δ 160.3, 156.6, 148.2, 147.7, 127.3, 127.1 (3C), 123.9, 113.5 (2C), 110.9, 108.9, 60.1, 55.9, 55.8, 55.5, 51.1, 43.7, 28.1; LCMS *m*/*z*: 382 [M + H]^+^. Elemental analysis: calcd. for C_20_H_23_N_5_O_3_ C 62.98, H 6.08, N 18.36%, found C 62.72, H 5.85, N 18.47%.

*4-Methoxy-5-[1-(4-methoxyphenyl)-1H-tetrazol-5-yl]-6-methyl-5,6,7,8-tetrahydro[1,3]dioxolo[4,5-g]isoquinoline* (**1b**), Yield 3.09 g (94%); beige solid; m.p. = 143–145 °С; *R*_f_ = 0.41 (EtOAc-hexane, 2:1); ^1^H-NMR (600 MHz, CDCl_3_): δ 7.55–7.52 (m, 2H, H-Ar), 7.06–7.03 (m, 2H, H-Ar), 6.34 (s, 1H, H-9), 5.85 (d, *J* = 1.4 Hz, 1H, 2-CH_2_), 5.81 (d, *J* = 1.4 Hz, 1H, 2-CH_2_), 5.04 (s, 1H, H-5), 3.89 (s, 3H, OCH_3_), 3.62 (s, 3H, OCH_3_), 3.24–3.19 (m, 1H, 7-CH_2_), 2.90–2.85 (m, 1H, 8-CH_2_), 2.68–2.65 (m, 1H, 7-CH_2_), 2.63–2.59 (m, 1H, 8-CH_2_), 2.22 (s, 3H, N–CH3); ^13^C-NMR (150 MHz, CDCl_3_): δ 160.9, 155.9, 149.0, 139.9, 134.1, 129.3, 127.4, 127.1 (2C), 117.2, 114.7 (2C), 103.2, 100.9, 58.9, 55.8, 52.7, 46.3, 42.1, 25.6; LCMS *m*/*z*: 396 [M + H]^+^. Elemental analysis: calcd. for C_20_H_21_N_5_O_4_ C 60.75, H 5.35, N 17.71%, found C 60.51, H 5.01, N 17.60%.

*1-[1-(4-Methoxyphenyl)-1H-tetrazol-5-yl]-2-methyl-1,2,3,4-tetrahydroisoquinoline* (**1c**), Yield 1.84 g (69%); yellow solid; m.p. = 90–92 °С; *R*_f_ = 0.59 (EtOAc–hexane, 1:2); ^1^H-NMR (600 MHz, CDCl_3_): δ 7.09 (t, *J* = 7.7 Hz, 1H, H-Ar), 7.03 (t, *J* = 7.4 Hz, 1H, H-Ar), 6.96 (d, *J* = 7.4 Hz, 1H, H-Ar), 6.93–6.91 (m, 2H, H-Ar), 6.77–6.75 (m, 2H, H-Ar), 6.72 (d, *J* = 7.7 Hz, 1H, H-Ar), 5.15 (s, 1H, H-1), 3.81 (s, 3H, OCH_3_), 3.00–2.95 (m, 1H, 3-CH_2_), 2.59–2.53 (m, 3H, 3,4-CH_2_), 2.29 (s, 3H, N–CH3); ^13^C-NMR (150 MHz, CDCl_3_): δ 160.4, 156.6, 134.8, 132.4, 128.6, 127.3 (2C), 127.1, 126.6 (2C), 126.2, 113.5 (2C), 60.3, 55.5, 51.1, 43.7, 28.5; LCMS *m*/*z*: 322 [M + H]^+^. Elemental analysis: calcd. for C_18_H_19_N_5_O C 67.27, H 5.96, N 21.79%, found C 66.95, H 6.20, N 21.91%.

### 3.2. The Interaction of Isoquinolines ***1a***, ***1b*** with Activated Alkynes (General Method)

Alkynes (DMAD, methyl propiolate or acetylacetylene) (4 mmol) was added to a solution of isoquinolines **1a** or **1b** (2 mmol) in trifluoroethanol (10 mL). The reaction mixture was kept at 20 °C, isoquinolines **1a**, **1b** reacted with methyl propiolate for 1 day, with acetylacetylene for 5 h and 10 days, respectively, with DMAD for 5 and 12 days, respectively. The reaction progress was monitored by TLC (sorbphil, EtOAc-hexane, 1:2). The solvent was evaporated in vacuum and the residue was recrystallized from EtOAc-hexane mixture.

*Methyl (4E)-8,9-dimethoxy-6-[1-(4-methoxyphenyl)-1H-tetrazol-5-yl]-3-methyl-1,2,3,6-tetrahydro-3-benzazocin-5-carboxylate* (**2a**), Yield 0.90 g (97%); white solid; m.p. = 152–154 °С; *R*_f_ = 0.41 (EtOAc-hexane, 2:1); IR (KBr) ν 1635 cm^−1^ (С=O); ^1^H-NMR (600 MHz, CDCl_3_): δ 7.43 (s, 1H, H-4), 7.08 (d, *J* = 8.7 Hz, 2H, H-Ar), 6.85 (d, *J* = 8.7 Hz, 2H, H-Ar), 6.57 (s, 1H, H-7), 6.32 (s, 1H, H-10), 6.00 (s, 1H, H-6), 3.98–3.93 (m, 1H, 2-CH_2_), 3.81 (s, 6H, OCH_3_), 3.66 (s, 3H, OCH_3_), 3.54 (s, 3H, OCH_3_), 3.06–2.96 (m, 3H, 1,2-CH_2_), 2.93 (s, 3H, N–CH3); ^13^C-NMR (150 MHz, CDCl_3_): δ 169.4, 160.8, 159.6, 153.6, 147.5, 147.3, 128.7, 127.9, 127.7 (2C), 126.6, 115.3, 114.6, 114.1 (2C), 94.0, 55.8, 55.6, 55.5, 51.4, 51.2, 44.2, 40.8, 35.1; LCMS *m*/*z*: 466 [M + H]^+^. Elemental analysis: calcd. for C_24_H_27_N_5_O_5_ C 61.92, H 5.85, N 15.04%, found C 61.70, H 6.01, N 14.90%.

*1-{(4E)-8,9-Dimethoxy-6-[1-(4-methoxyphenyl)-1H-tetrazol-5-yl]-3-methyl-1,2,3,6-tetrahydro-3-benzazocine-5-yl}ethanone* (**2b**), Yield 0.78 g (87%); beige solid; m.p. = 194–195 °С; *R*_f_ = 0.26 (EtOAc- hexane, 2:1); IR (KBr) ν 1620 cm^−1^ (С=O); ^1^H-NMR (600 MHz, CDCl_3_): δ 7.32 (s, 1H, H-4), 7.10 (d, *J* = 9.0 Hz, 2H, H-Ar), 6.84 (d, *J* = 9.0 Hz, 2H, H-Ar,), 6.53 (s, 1H, H-7), 6.40 (s, 1H, H-10), 6.25 (s, 1H, H-6), 4.24–4.19 (m, 1H, 2-CH_2_), 3.81 (s, 6H, OCH_3_), 3.63 (s, 3H, OCH_3_), 3.10 (ddd, *J* = 15.5, 6.6, 2.7 Hz, 1H, 2-CH_2_), 3.01 (s, 3H, N–CH3), 2.98–2.93 (m, 2H, 1-CH_2_), 2.07 (s, 3H, COCH_3_); ^13^C-NMR (150 MHz, CDCl_3_): δ 193.3; 160.7, 159.5, 155.8, 147.4, 147.3, 128.4, 128.0, 127.6 (2C), 126.6, 115.5, 114.5, 114.0 (2C), 108.5, 55.8, 55.54, 55.51, 51.2, 44.4, 38.0, 34.7, 24.7; LCMS *m*/*z*: 450 [M + H]^+^. Elemental analysis: calcd. for C_24_H_27_N_5_O_4_ C 64.13, H 6.05, N 15.58%, found C 63.92, H 6.25, N 15.41%.

*Dimethyl (4E)-8,9-dimethoxy-6-[1-(4-methoxyphenyl)-1H-tetrazol-5-yl]-3-methyl-1,2,3,6-tetrahydro-3-benzazocin-4,5-dicarboxylate* (**2c**), Yield 0.97 g (93%); beige solid; m.p. = 108–110 °С; *R*_f_ = 0.47 (EtOAc-hexane, 1:1); IR (KBr) ν 1736, 1683 cm^−1^ (С=O); ^1^H-NMR (600 MHz, CDCl_3_): δ 6.98 (d, *J* = 8.7 Hz, 2H, H-Ar), 6.81 (d, *J* = 8.7 Hz, 2H, H-Ar), 6.48 (s, 1H, H-10), 6.14 (s, 1H, H-7), 5.88 (s, 1H, H-6), 4.65–4.62 (m, 1H, 2-CH_2_), 3.80 (s, 3H, OCH_3_), 3.79 (s, 3H, OCH_3_), 3.72 (s, 3H, OCH_3_), 3.61 (s, 3H, OCH_3_), 3.60 (s, 3H, OCH_3_), 3.35 (ddd, *J* = 15.1, 8.7, 1.8 Hz, 1H, 1-CH_2_), 2.86 (ddd, *J* = 8.7, 11.4, 16.2 Hz, 1H, 2-CH_2_), 2.56–2.53 (m, 1H, 1-CH_2_), 2.52 (s, 3H, N–CH3); ^13^C-NMR (150 MHz, CDCl_3_): δ 168.2, 166.2, 160.8, 157.8, 156.7, 147.9, 147.4, 129.6, 127.4 (2C), 126.4, 126.2, 115.8, 114.2 (2C), 113.8, 99.8, 55.9, 55.6, 55.5, 55.2, 52.5, 51.8, 43.2, 38.3, 32.8; LCMS *m*/*z*: 524 [M + H]^+^. Elemental analysis: calcd. for C_26_H_29_N_5_O_7_ C 59.65, H 5.58, N 13.38%, found C 59.83, H 5.71, N 13.45%.

*Methyl (8E)-10-[1-(4-methoxyphenyl)-1H-tetrazol-5-yl]-7,11-dimethyl-5,6,7,10-tetrahydro[1,3]dioxolo[4,5-i][3]benzazocine-9-carboxylate* (**2d**), Yield 0.93 g (97%); white solid; m.p. = 167–169 °С; *R*_f_ = 0.35 (EtOAc-hexane, 2:1); IR (KBr) ν 1681 cm^−1^ (С=O); ^1^H-NMR (600 MHz, CDCl_3_): δ 7.42 (m, 1H, H-8), 7.16 (d, *J* = 8.8 Hz, 2H, H-Ar), 6.90 (d, *J* = 8.8 Hz, 2H, H-Ar), 6.85 (s, 1H, H-10), 6.30 (s, 1H, H-4), 5.88 (d, *J* = 1.5 Hz, 1H, 2-CH_2_), 5.83 (d, *J* = 1.5 Hz, 1H, 2-CH_2_), 4.03 (ddd, *J* = 15.3, 9.8, 4.8 Hz, 1H, 6-CH_2_), 3.84 (s, 3H, OCH_3_), 3.83 (s, 3H, OCH_3_), 3.55 (s, 3H, OCH_3_), 3.08 (dt, *J* = 15.3, 5.3 Hz, 1H, 6-CH_2_), 2.92 (s, 3H, N–CH3), 2.90–2.89 (m, 1H, 5-CH_2_), 2.80 (dt, *J* = 16.3, 5.3 Hz, 1H, 5-CH_2_); ^13^C-NMR (150 MHz, CDCl_3_): δ 167.0, 160.6, 160.0, 153.5, 147.8, 141.2, 135.6, 132.3, 127.1 (2C), 126.9, 121.6, 113.9 (2C), 105.3, 101.0, 99.3, 59.9, 55.6, 52.4, 51.1, 45.0, 34.9, 30.0; LCMS *m*/*z*: 480 [M + H]^+^. Elemental analysis: calcd. for C_24_H_25_N_5_O_6_ C 60.12, H 5.26, N 14.61%, found C 59.89, H 5.46, N 14.52%.

*1-{(8E)-11-Methoxy-10-[1-(4-methoxyphenyl)-1H-tetrazol-5-yl]-7-methyl-5,6,7,10-tetrahydro[1,3]dioxolo[4,5-i][3]benzazocin-9-yl}ethanone* (**2e**), Yield 0.88 g (95%); white solid; m.p. = 225–227 °С; *R*_f_ = 0.42 (EtOAc-hexane, 1:1); IR (KBr) ν 1620 cm^−1^ (С=O); ^1^H-NMR (600 MHz, CDCl_3_): δ 7.27 (s, 1H, H-8); 7.18 (br.s, 1H, H-10), 7.15–7.12 (m, 2H, H-Ar), 6.87–6.85 (m, 2H, H-Ar), 6.28 (s, 1H, H-4), 5.87 (d, *J* = 1.4 Hz, 1H, 2-CH_2_), 5.81 (d, *J* = 1.4 Hz, 1H, 2-CH_2_), 4.15–4.10 (m, 1H, 6-CH_2_), 3.83 (s, 3H, OCH_3_), 3.80 (s, 3H, OCH_3_), 3.10 (ddd, *J* = 15.6, 6.4, 4.6 Hz, 1H, 6-CH_2_), 2.96 (s, H, N–CH3), 2.87 (ddd, *J* = 16.6, 10.2, 6.4 Hz, 1H, 5-CH_2_), 2.67 (dt, *J* = 16.6, 4.6 Hz, 1H, 5-CH_2_), 2.06 (s, 3H, CH_3_); ^13^C-NMR (150 MHz, CDCl_3_): δ 192.7; 160.3, 159.7, 155.3, 147.6, 141.1, 135.4, 131.6, 126.7, 126.7 (2C), 121.4, 113.7 (2C), 107.7, 104.9, 100.7, 59.6, 55.4, 52.1, 44.7, 34.5, 27.9, 24.5; LCMS *m*/*z*: 464 [M + H]^+^. Elemental analysis: calcd. for C_24_H_25_N_5_O_5_ C 62.19, H 5.44, N 15.11%, found C 61.93, H 5.63, N 15.26%.

*Dimethyl (8E)-11-methoxy-10-[1-(4-methoxyphenyl)-1H-tetrazol-5-yl]-7-methyl-5,6,7,10-tetrahydro[1,3]dioxolo[4,5-i][3]benzazocin-8,9-dicarboxylate* (**2f**), Yield 1.03 g (96%); white solid; m.p. = 212–214 °С; *R*_f_ = 0.47 (EtOAc-hexane, 1:1); IR (KBr) ν 1732, 1691 cm^−1^ (С=O); ^1^H-NMR (600 MHz, CDCl_3_): δ 7.13–7.12 (m, 2H, H-Ar), 6.86–6.84 (m, 2H, H-Ar), 6.72 (s, 1H, H-10), 6.22 (s, 1H, H-4), 5.86 (d, *J* = 1.4 Hz, 1H, 2-CH_2_), 5.78 (d, *J* = 1.4 Hz, 1H, 2-CH_2_), 4.35 (ddd, *J* = 15.2, 10.0, 6.0 Hz, 1H, 6-CH_2_), 3.81 (s, 3H, OCH_3_), 3.75 (s, 3H, OCH_3_), 3.72 (s, 3H, OCH_3_), 3.58 (s, 3H, OCH_3_), 3.30 (ddd, *J* = 15.2, 6.7, 4.0 Hz, 1H, 6-CH_2_), 2.87 (ddd, *J* = 16.7, 10.0, 6.7 Hz, 1H, 5-CH_2_), 2.53 (s, 3H, N–CH3), 2.45–2.41 (m, 1H, 5-CH_2_); ^13^C-NMR (150 MHz, CDCl_3_): δ 167.5; 166.4, 160.6, 158.3, 155.6, 148.1, 141.1, 135.7, 132.6, 126.8 (2C), 126.5, 120.4, 114.0 (2C), 105.1, 101.1, 99.7, 60.1, 55.9, 55.6, 52.4, 51.7, 38.8, 32.62, 32.60; LCMS *m*/*z*: 538 [M + H]^+^. Elemental analysis: calcd. for C_26_H_27_N_5_O_8_ C 58.10, H 5.06, N 13.03%, found C 57.83, H 5.21, N 12.90%.

### 3.3. The Interaction of Isoquinoline ***1c*** with DMAD

DMAD (0.44 g, 3.1 mmol) was added to a solution of isoquinoline **1c** (0.5 g, 1.56 mmol) in trifluoroethanol (5 mL). The mixture was kept at 20 °C for 2 days. The reaction progress was monitored by TLC (sorbfil, EtOAc-hexane, 1:3). The solvent was evaporated in vacuum, and the residue was chromatographed on a silica gel column. Azocine **2g** and spiro compound **3** were eluted with EtOAc-hexane, 1:2, and recrystallized from EtOAc-hexane mixture.

*Dimethyl 6-[1-(4-methoxyphenyl)-1H-tetrazol-5-yl]-3-methyl-1,2,3,6-tetrahydro-3-benzazocin-4,5-dicarboxylate* (**2g**), Yield 0.36 g (50%); white solid; m.p. = 125–127 °С; *R*_f_ = 0.70 (EtOAc-hexane, 1:2); IR (KBr) ν 1734, 1681 cm^−1^ (С=O); ^1^H-NMR (600 MHz, CDCl_3_): δ 7.10–7.08 (m, 1H, H-Ar); 7.02–6.97 (m, 4H, H-Ar), 6.81–6.80 (m, 3H, H-Ar), 6.03 (s, 1H, H-6), 4.60–4.53 (m, 1H, 2-CH_2_), 3.82 (s, 3H, OCH_3_), 3.73 (s, 3H, OCH_3_), 3.63 (s, 3H, OCH_3_), 3.39 (dd, *J* = 13.8, 8.1 Hz, 1H, 2-CH_2_,), 2.99–2.93 (m, 1H, 1-CH_2_), 2.62 (dd, *J* = 15.9, 6.8 Hz, 1H, 1-CH_2_,), 2.49 (s, 3H, N–CH3); ^13^C-NMR (150 MHz, DMSO-*d_6_*): δ 167.4, 165.6, 160.3, 157.6, 155.8, 137.2, 134.2, 132.3, 130.9, 127.5, 127.1 (2С), 127.0, 126.0, 114.3 (2С), 99.4, 55.6, 54.0, 52.4, 51.6, 42.6, 38.2, 32.6; LCMS *m*/*z*: 464 [M + H]^+^. Elemental analysis: calcd. for C_24_H_25_N_5_O_5_ C 62.19, H 5.44, N 15.11%, found C 61.93, H 5.69, N 15.28%.

*Dimethyl (11E)-11-{[1-(4-methoxyphenyl)-1H-tetrazol-5-yl]methylidene}-3-methyl-3-azaspiro[5.5]undeca-1,7,9-triene-1,2-dicarboxylate* (**3**), Yield 0.12 g (17%); white solid; m.p. = 126–127 °С; *R*_f_ = 0.50 (EtOAc-hexane, 1:1); IR (KBr) ν 1736, 1680 cm^−1^ (С=O); ^1^H-NMR (600 MHz, CDCl_3_): δ 7.73 (d, *J* = 9.7 Hz, 1H, H-cyclohexadiene); 7.34 (d, *J* = 8.7 Hz, 2H, H-Ar), 7.04 (d, *J* = 8.7 Hz, 2H, H-Ar), 6.31 (dd, *J* = 9.7, 5.5 Hz, 1H, H-cyclohexadiene), 6.03–5.99 (m, 2H, H-cyclohexadiene), 5.91 (s, 1H, =CH-Ar), 3.88 (s, 3H, OCH_3_), 3.83 (s, 3H, OCH_3_), 3.54 (s, 3H, OCH_3_), 3.22 (ddd, *J* = 13.1, 9.6, 3.9 Hz, 1H, 4-CH_2_), 2.97 (ddd, *J* = 13.1, 4.8, 4.6 Hz, 1H, 4-CH_2_), 2.75 (s, 3H, N–CH3), 1.87 (ddd, *J* = 13.6, 9.6, 3.9 Hz, 1H, 5-CH_2_,), 1.81–1.77 (m, 1H, 5-CH_2_); ^13^C-NMR (150 MHz, CDCl_3_): δ 166.6, 165.6, 160.5, 156.4, 151.2, 150.7, 128.5, 142.0, 126.7, 126.2 (2С), 122.0, 119.4, 114.8 (2С), 105.3, 97.4, 55.6, 52.6, 50.9, 42.5 (2С), 39.5, 33.7, LCMS *m*/*z*: 464 [M + H]^+^. Elemental analysis: calcd. for C_24_H_25_N_5_O_5_ C 62.19, H 5.44, N 15.11%, found C 61.43, H 5.72, N 15.21%.

### 3.4. X-ray Structure Determination of Compound ***3***

The structures of product **3** were unambiguously established by X-ray diffraction study and are shown in Figure 1 along with the atomic numbering schemes. The tetrahydropyridine ring of the spiro compound **3** assumes a slightly distorted “sofa” conformation with the carbon atom C(16) extending from the plane formed by the remaining atoms of the ring by 0.658 Å. Carbonyl fragment C(19)–O(3) of the ester group is practically coplanar with the basal plane of the tetrahydropyridine ring C(15)–C(18)=C(21)–N(5)–C(17) (the dihedral angle is equal to 6.40°) due to the conjugation of bonds. The nitrogen atom N(5) has a trigonal planar configuration (the sum of the valence angles is equal to 359.7°). The cyclohexadiene ring of the spiro compound **3** assumes a slightly distorted “sofa” conformation with the carbon atom C(15) extending from the plane formed by the remaining atoms of the ring by 0.444 Å. The 4-methoxyphenyl substituent in molecule **3** is twisted with a tetrazole ring at an angle of 34.20°. The molecule of spiro compound **3** contains the asymmetric carbon atom C(15). The molecules of compound **3** in the crystal form centrosymmetric dimers due to two intermolecular hydrogen bonds C(5)–H(5)···O(5)*. 

The crystals of compound **3** (C_24_H_25_N_5_O_5_, *M* 463.49) are triclinic, space group *P*-1 at 120 K: *a* = 8.2773(9), *b* = 11.6273(13), *c* = 11.9759(12) Å; β = 94.980(2)°; *V* 1097.7(2) Å^3^; *Z* 2; *d*_calc_ 1.402 g cm^−3^; *F*(000) 488.0; μ 0.101 mm^−1^. The unit cell parameters and intensity of 8547 reflections (4994 independent reflections, *R*_int_ 0.0235) were measured on an automatic three-circle diffractometer Bruker SMART APEX-II CCD (MoKa radiation (*λ* = 0.71073 Å), graphite-monochromator, ω-scanning, 3.57° ≤ 2Θ ≤ 54.968°).

The structure was determined by the direct method and refined by the least-squares technique in the full-matrix anisotropic approximation for non-hydrogen atoms based on *F*^2^. The positions of hydrogen atoms were calculated geometrically and were included in the refinement with fixed positional parameters (the “rider” model) and with isotropic displacement parameters (*U*_iso_(H) = 1.5 U_eq_(C) for CH_3_ groups and 1.2*U*_eq_(C) for other groups). The final probability factors were *R*_1_ = 0.044 (*I* ≥ 2*σ* (*I*)) and *wR*_2_ = 0.106 for all independent reflections. All calculations were carried out using the programs OLEX-2 [20] and SHELXTL [21] software package. Tables of the coordinates of atoms, bond lengths, valence and torsion angles, and anisotropic temperature parameters of compound **3** were deposited at the Cambridge Crystallographic Data Center (deposit CCDC 1848342).

### 3.5. The Interaction of Isoquinoline ***1c*** with Methyl Propiolate

Methyl propiolate (0.52 g, 6.2 mmol) was added to a solution of isoquinoline **1c** (0.5 g, 1.56 mmol) in trifluoroethanol (7 mL). The reaction mixture was heated under reflux for 120 h. The reaction progress was monitored by TLC (sorbfil, EtOAc-hexane, 1:3). The solvent was evaporated in vacuum. The residue was chromatographed on a silica gel column. Vinylisoquinoline **4a** and benzazocine **2h** were eluted with EtOAc-hexane, 1:2, and recrystallized from EtOAc-hexane mixture.

*Methyl (4E)-6-[1-(4-methoxyphenyl)-1H-tetrazol-5-yl]-3-methyl-1,2,3,6-tetrahydro-3-benzazocin-5-carboxylate* (**2h**), Yiled 0.14 g (22%); white solid; m.p. = 139–140 °С; *R*_f_ = 0.37 (EtOAc-hexane, 1:1); IR (KBr) ν 1679 cm^−1^ (С=O); ^1^H-NMR (600 MHz, CDCl_3_): δ 7.37 (s, 1H, H-4), 7.13–7.10 (m, 4H, H-Ar), 7.05–7.02 (m, 1H, H-Ar), 6.96 (d, *J* = 7.8 Hz, 1H, H-Ar), 6.86 (d, *J* = 8.7 Hz, 2H, H-Ar), 6.13 (s, 1H, H-6), 3.88–3.84 (m, 1H, 2-CH_2_), 3.83 (s, 3H, OCH_3_), 3.53 (s, 3H, OCH_3_), 3.13–3.03 (m, 3H, 1,2-CH_2_), 2.91 (s, 3H, N–CH3); ^13^C-NMR (150 MHz, CDCl_3_): δ 169.3, 160.8, 159.4, 153.6, 136.6, 136.1, 132.4, 131.8, 127.7 (2С), 127.32, 127.26, 126.7, 114.1 (2С), 94.3, 55.6, 51.32, 51.25, 44.2, 41.3, 35.7; LCMS *m*/*z*: 406 [M + H]^+^. Elemental analysis: calcd. for C_22_H_23_N_5_O_3_ C 65.17, H 5.72, N 17.27%, found C 65.36, H 5.95, N 17.03%.

*Methyl (2E)-3-{1-[1-(4-methoxyphenyl)-1H-tetrazol-5-yl]-2-methyl-1,2,3,4-tetrahydroisoquinolin-1-yl}prop-2-enoate* (**4а**), Yield 0.063 g (10%); white solid; m.p. = 132–134 °С; *R*_f_ = 0.54 (EtOAc-hexane, 1:2); IR (KBr) ν 1681 cm^−1^ (С=O); ^1^H-NMR (600 MHz, CDCl_3_): δ 7.84 (d, *J* = 16.0 Hz, 1H, CH=CHCO_2_CH_3_), 7.11–7.09 (m, 1H, H-Ar), 7.01 (t, *J* = 7.5 Hz, 1H, H-Ar), 6.90 (d, *J* = 7.5 Hz, 1H, H-Ar), 6.66 (d, *J* = 8.7 Hz, 2H, H-Ar), 6.58–6.55 (m, 3H, H-Ar), 5.36 (d, *J* = 16.0 Hz, 1H, CH=CHCO_2_CH_3_), 3.77 (s, 3H, OCH_3_), 3.73 (s, 3H, OCH_3_), 2.64–2.59 (m, 1H, 3-CH_2_), 2.52 (dd, *J* = 12.6, 5.1 Hz, 1H, 4-CH_2_), 2.42–2.40 (m, 1H, 3-CH_2_), 2.24–2.20 (m, 1H, 4-CH_2_), 2.18 (s, 3H, N–CH3); ^13^C-NMR (150 MHz, CDCl_3_): δ 165.8, 160.3, 158.5, 144.5, 135.5, 133.3, 128.7 (2C), 127.8, 127.51 (2C), 127.46, 126.6, 126.2, 113.2 (2C), 63.4, 55.5, 51.7, 45.5, 38.8, 28.5; LCMS *m*/*z*: 406 [M + H]^+^. Elemental analysis: calcd. for C_22_H_23_N_5_O_3_ C 65.17, H 5.72, N 17.27%, found C 64.42, H 5.89, N 17.41%.

### 3.6. The Interaction of Isoquinoline ***1c*** with Acetylacetylene

Acetylacetylene (0.54 g, 7.9 mmol) was added to a solution of isoquinoline **1c** (0.25 g, 0.78 mmol) in trifluoroethanol (5 mL). The reaction mixture was refluxed for 180 hours. The reaction progress was monitored by TLC (sorbfil, EtOAc-hexane, 1:3). The solvent was evaporated in vacuum. The residue was purified by column chromatography on silica gel. Isoquinoline **4b** was eluted with EtOAc-hexane, 1:3.

*(3E)-4-{1-[1-(4-Methoxyphenyl)-1H-tetrazol-5-yl]-2-methyl-1,2,3,4-tetrahydroisoquinolin-1-yl}but-3-ene-2-one* (**4b**), Yiled 0.052 g (17%); white solid; m.p. = 132–134 °С; *R*_f_ = 0.40 (EtOAc-hexane, 1:2); IR (KBr) ν 1630 cm^−1^ (С=O); ^1^H-NMR (600 MHz, CDCl_3_): *δ* 7.73 (d, *J* = 16.5 Hz, 1H, CH=CH-Ac), 7.13 (t, *J* = 7.5 Hz, 1H, H-Ar), 7.03 (t, *J* = 7.9 Hz, 1H, H-Ar), 6.92 (d, *J* = 7.5 Hz, 1H, H-Ar), 6.69 (d, *J* = 9.1 Hz, 2H, H-Ar), 6.63 (d, *J* = 9.1 Hz, 2H, H-Ar), 6.59 (d, *J* = 7.9 Hz, 1H, H-Ar), 5.58 (d, *J* = 16.5 Hz, 1H, CH=CH-Ac), 3.80 (s, 3H, OCH_3_), 2.73–2.64 (m, 1H, 3-CH_2_), 2.60–2.54 (m, 1H, 4-CH_2_), 2.49–2.46 (m, 1H, 3-CH_2_), 2.39 (m, 3H, N–CH3), 2.31–2.20 (m, 4H, COCH_3_ and 4-CH_2_); ^13^C-NMR (150 MHz, CDCl_3_): *δ* 198.3, 160.5, 158.2, 143.7, 136.2, 135.5, 133.2, 128.9, 128.8, 128.1, 127.7 (2C), 127.5, 126.5, 113.4 (2C), 63.7, 55.7, 45.8, 39.0, 28.5, 27.1; LCMS *m*/*z*: 390 [M + H]^+^. Elemental analysis: calcd. for C_22_H_23_N_5_O_2_ C 67.85, H 5.95, N 17.98%, found C 67.98, H 6.21, N 18.10%.

## Supplementary Materials

The following are available online. ^1^H and ^13^C-NMR spectra of all the products **1**–**4** Figures S1–S29.
