# Supplementary material for: Synthesis of 1-(para-methoxyphenyl)tetrazolyl-Substituted 1,2,3,4-Tetrahydroisoquinolines and Their Transformations Involving Activated Alkynes
Source: Molecules. 2018 Nov 17;23(11):3010. doi: 10.3390/molecules23113010 (PMC6278526; doi:10.3390/molecules23113010)
Supplement: Supplementary file 1 [file molecules-23-03010-s001.pdf]

Synthesis of 1-(*para*-methoxyphenyl)tetrazolyl-Substituted 1,2,3,4-Tetrahydroisoquinolines and Their Transformations Involving Activated Alkynes

Alexander A. Titov [1], Reza Samavati [1], Elena V. Alexandrova [1], Tatiana N. Borisova [1], Tuyet Anh Dang Thi [2], Van Tuyen Nguyen [2,3], Tuan Anh Le [4], Alexey V. Varlamov [1], Erik V. Van der Eycken [1,5] and Leonid G. Voskressensky [1,*]

1 Peoples' Friendship University of Russia (RUDN University), 6 Miklukho-Maklaya St., 117198 Moscow, Russia; titov_aa@pfur.ru (A.A.T.); reza_nasa3@yahoo.com (R.S.); elena-aleksandrova-00-lena@mail.ru (E.V.A.); borisova_tn@pfur.ru (T.N.B.); avarlamov@sci.pfu.edu.ru (A.V.V.); erik.vandereycken@kuleuven.be (E.V.V.E.)
2 Institute of Chemistry of Vietnam Academy of Science and Technology, 18 Hoang Quoc Viet, Cau Giay, 100000 Hanoi, Vietnam; dangtuyetanh1201@gmail.com (T.A.D.T.); ngvtuyen@hotmail.com (V.T.N.)
3 Graduate University of Science and Technology, Vietnam Academy of Science and Technology, 18 Hoang Quoc Viet, Cau Giay, 100000 Hanoi, Vietnam
4 Faculty of Chemistry of VNU University of Science, 19 Le Thanh Tong, Hoan Kiem, 100000 Hanoi, Vietnam; huschemical.lab@gmail.com (L.T.A.)
5 Laboratory for Organic & Microwave-Assisted Chemistry (LOMAC), Department of Chemistry, KU Leuven Celestijnenlaan 200F, 3001 Leuven, Belgium
* Correspondence: lvoskressensky@sci.pfu.edu.ru; Tel.: +7-495-955-0729

CONTENTS

X-ray structure ……………....……………………………………………………..……...**S1**

Copies of ^{1}H and ^{13}C NMR spectra………………….……………………………….…………**S2-S29**

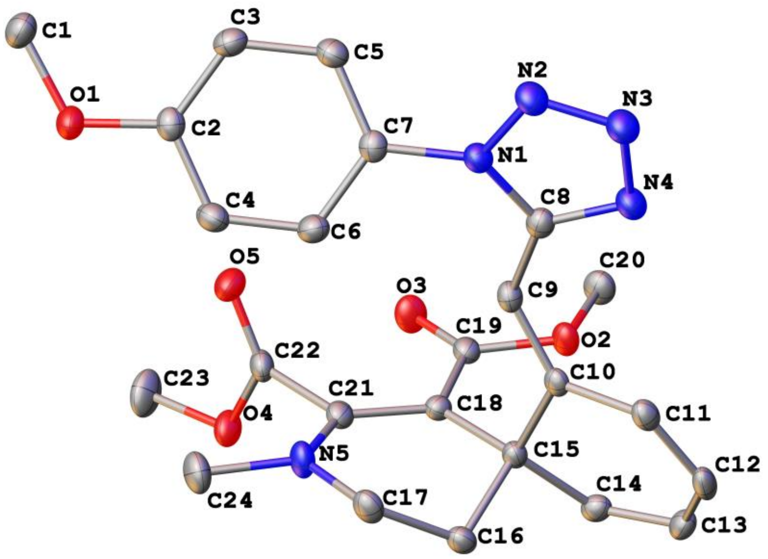

Figure S1.The X-ray crystal diffraction of compound **3** (deposit CCDC 1848342).

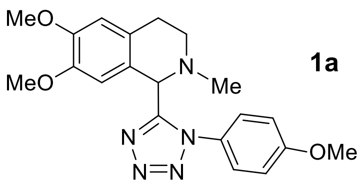

1a

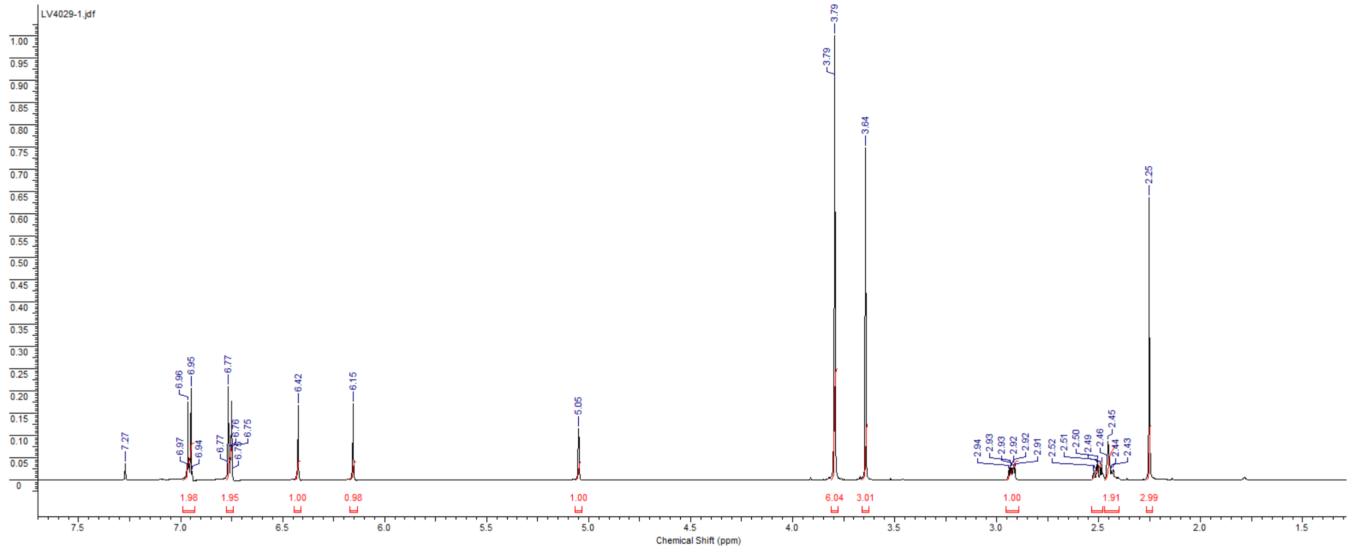

Figure S2. ^{1}H NMR spectrum of 6,7-dimethoxy-1-[1-(4-methoxyphenyl)-1*H*-tetrazol-5-yl]-2-methyl-1,2,3,4-tetrahydroisoquinoline **1a.**

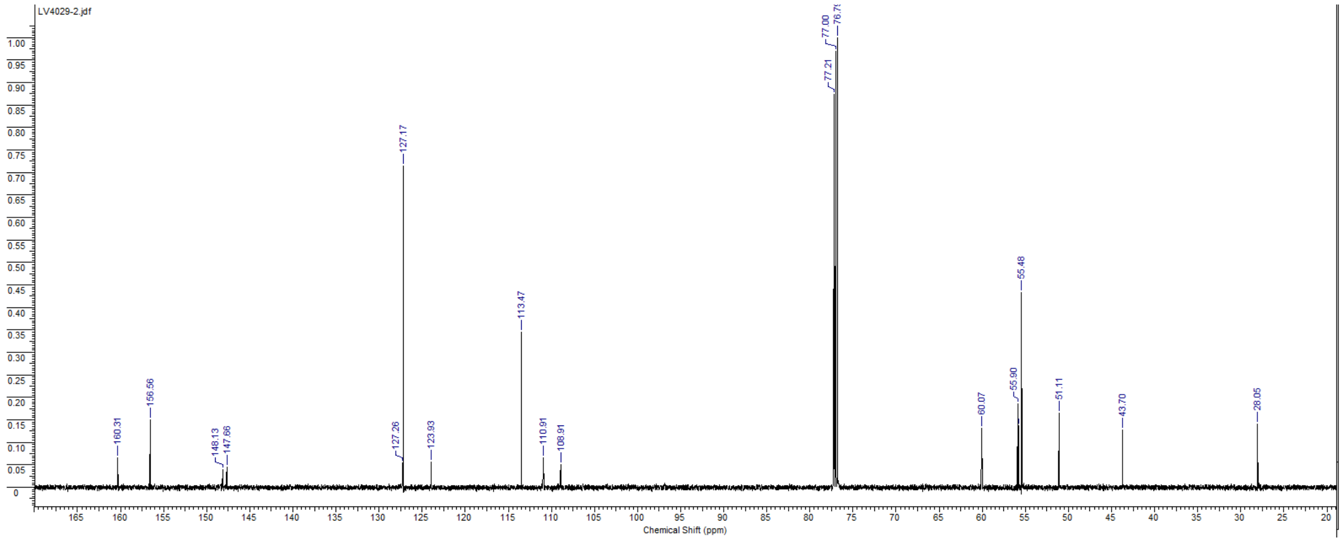

Figure S3. ^{13}C NMR spectrum of 6,7-dimethoxy-1-[1-(4-methoxyphenyl)-1*H*-tetrazol-5-yl]-2-methyl-1,2,3,4-tetrahydroisoquinoline **1a.**

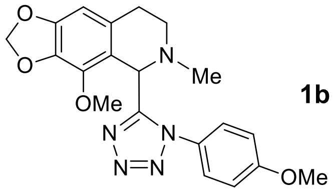

1b

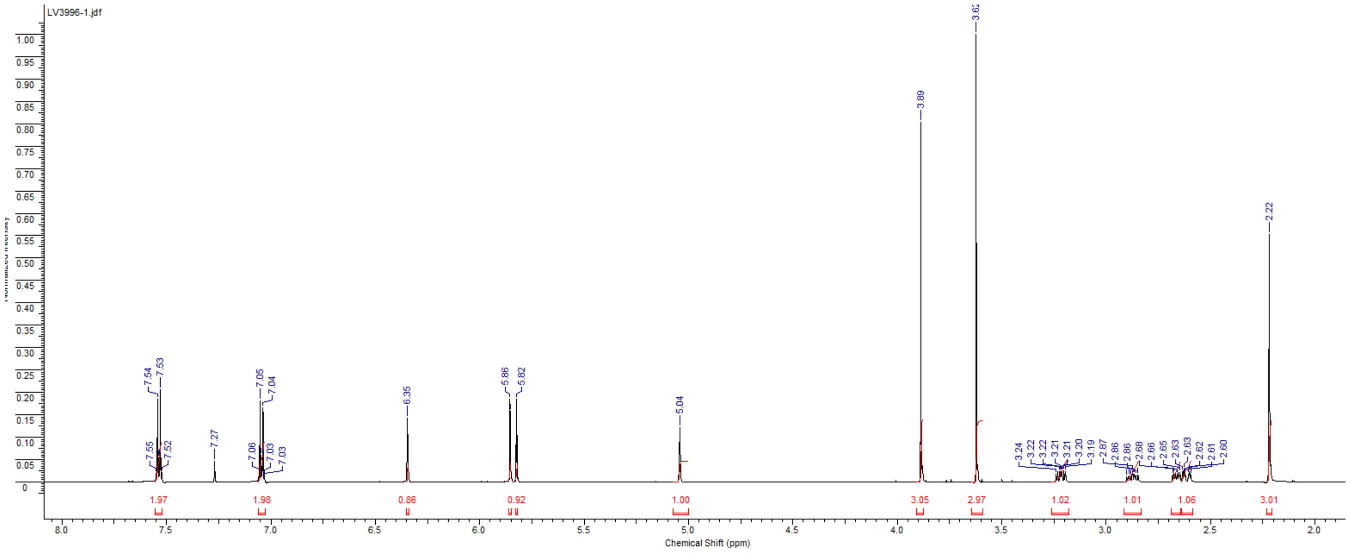

Figure S4. ^{1}H NMR spectrum of 4-methoxy-5-[1-(4-methoxyphenyl)-1*H*-tetrazol-5-yl]-6-methyl-5,6,7,8-tetrahydro[1,3]dioxolo[4,5-*g*]isoquinoline **1b.**

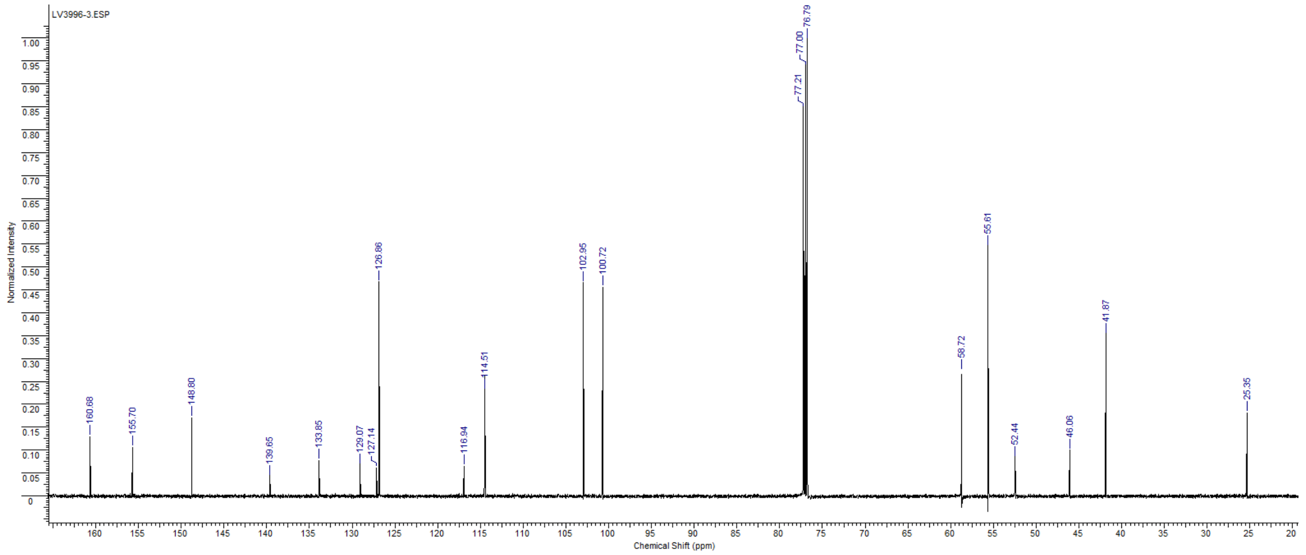

Figure S5. ^{13}C NMR spectrum of 4-methoxy-5-[1-(4-methoxyphenyl)-1*H*-tetrazol-5-yl]-6-methyl-5,6,7,8-tetrahydro[1,3]dioxolo[4,5-*g*]isoquinoline **1b.**

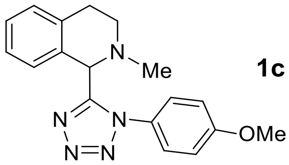

1c

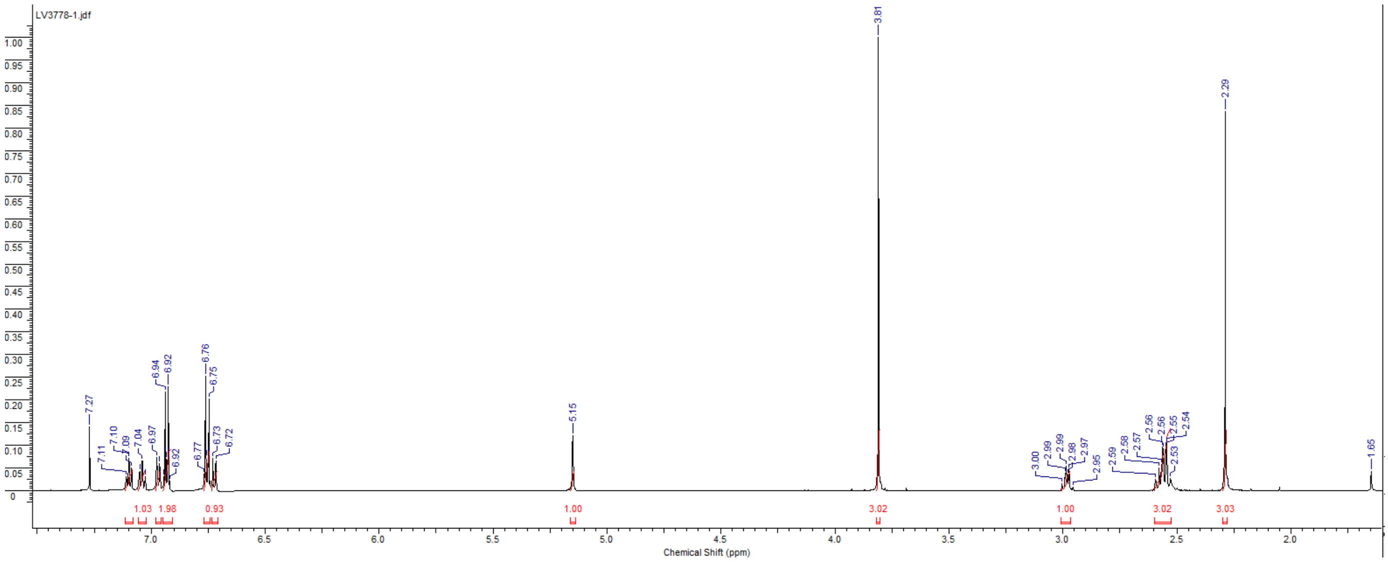

Figure S6. ^{1}H NMR spectrum of 1-[1-(4-methoxyphenyl)-1*H*-tetrazol-5-yl]-2-methyl-1,2,3,4-tetrahydroisoquinoline **1c**.

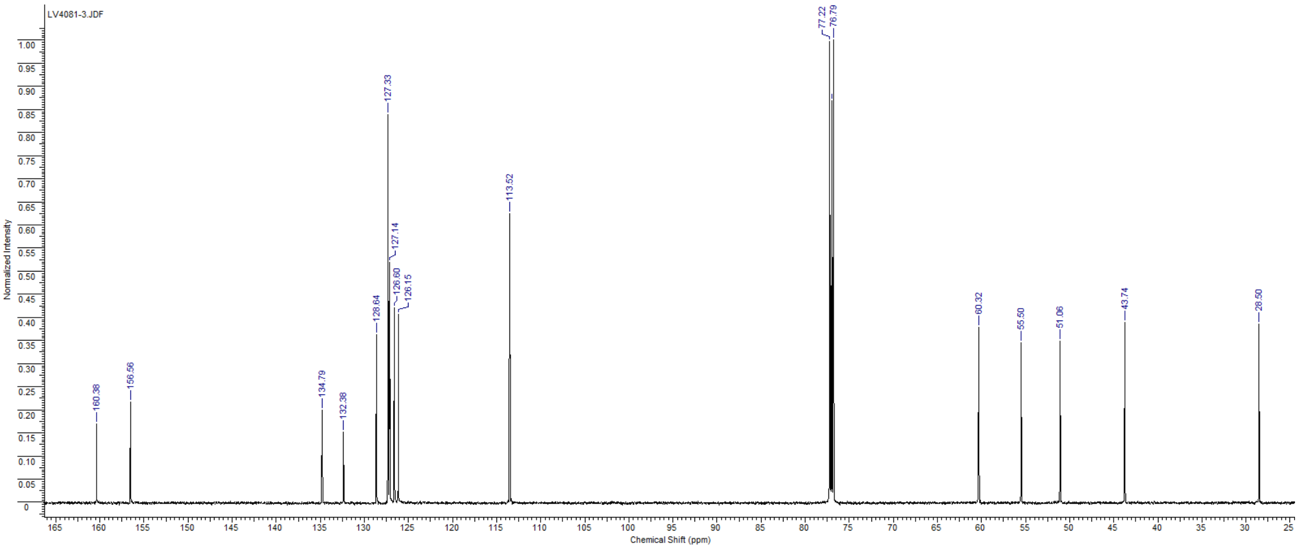

Figure S7. ^{13}C NMR spectrum of 1-[1-(4-methoxyphenyl)-1*H*-tetrazol-5-yl]-2-methyl-1,2,3,4-tetrahydroisoquinoline **1c.**

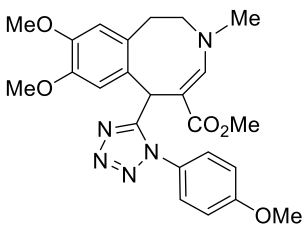

2a

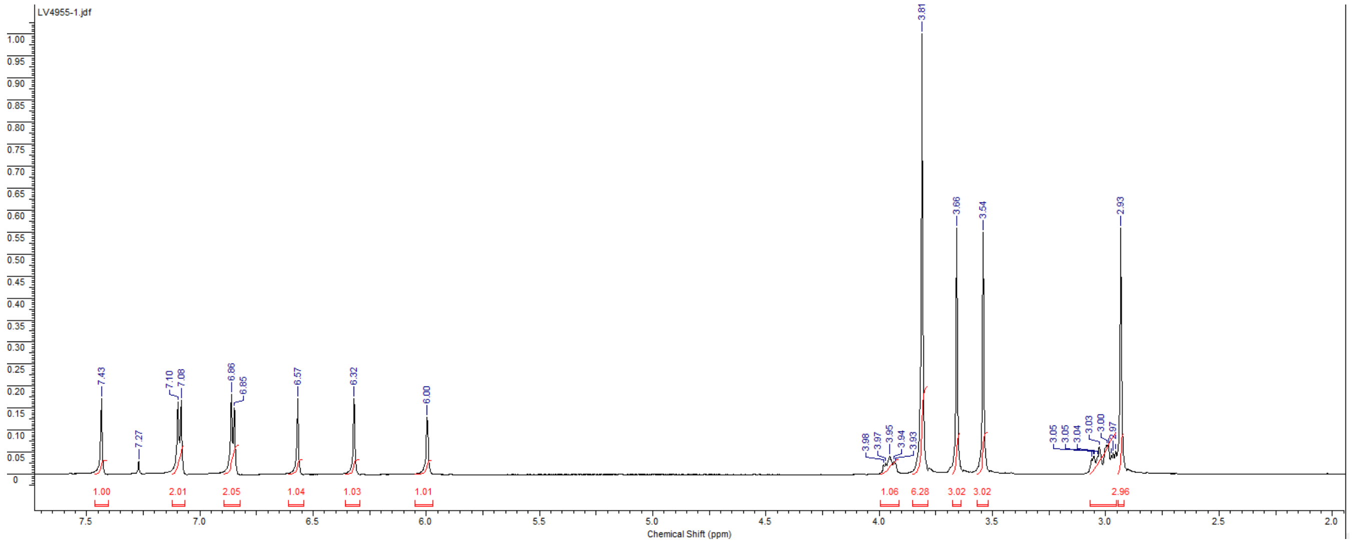

Figure S8. ^{1}H NMR spectrum of methyl (4*E*)-8,9-dimethoxy-6-[1-(4-methoxyphenyl) -1*H*-tetrazol-5-yl]-3-methyl-1,2,3,6-tetrahydro-3-benzazocin-5-carboxylate **2a.**

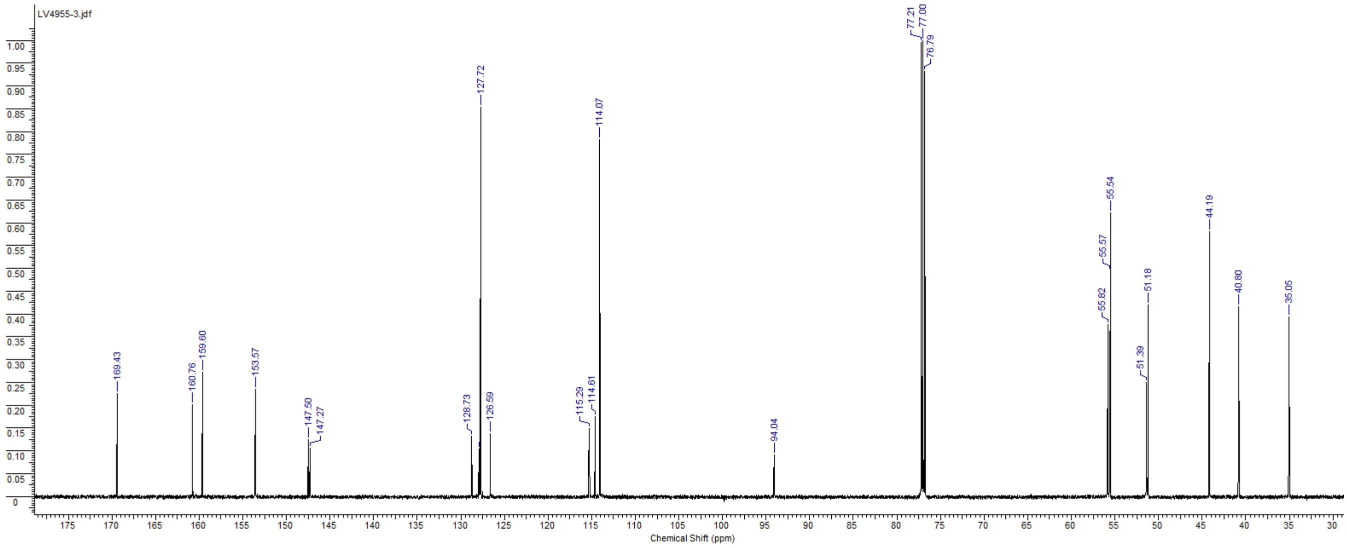

Figure S9. ^{13}C NMR spectrum of methyl (4*E*)-8,9-dimethoxy-6-[1-(4-methoxyphenyl) -1*H*-tetrazol-5-yl]-3-methyl-1,2,3,6-tetrahydro-3-benzazocin-5-carboxylate **2a.**

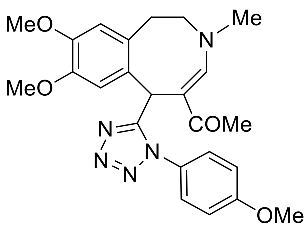

2b

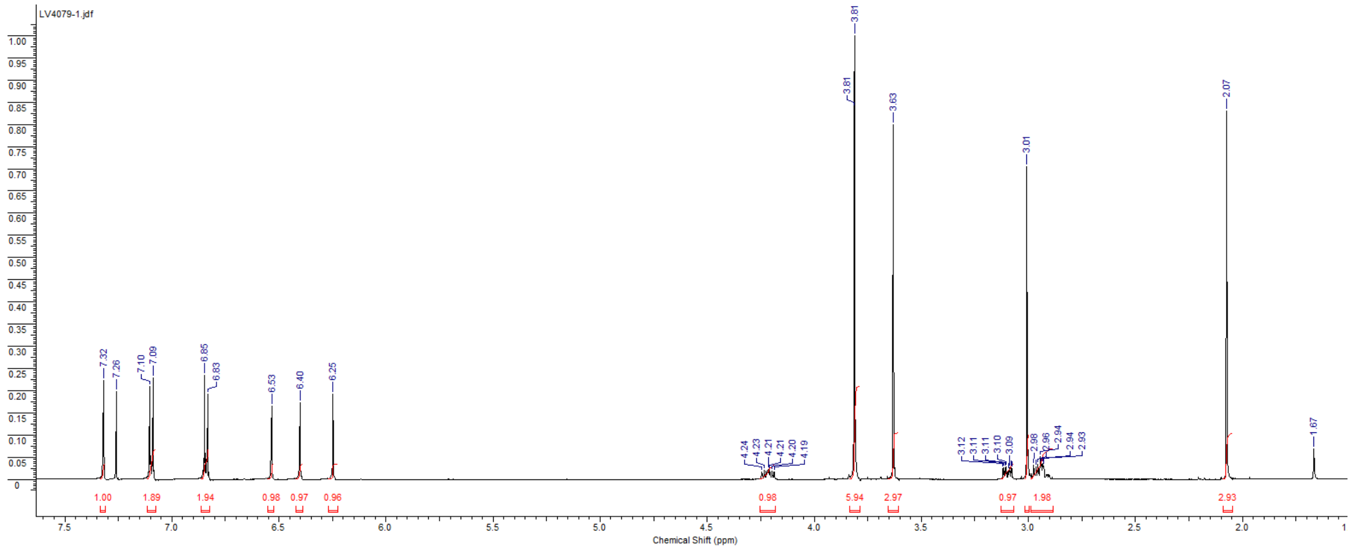

Figure S10. ^{1}H NMR spectrum of 1-{(4*E*)-8,9-dimethoxy-6-[1-(4-methoxyphenyl)-1*H*-tetrazol-5-yl]-3-methyl-1,2,3,6-tetrahydro-3-benzazocine-5-yl}-ethanone **2b.**

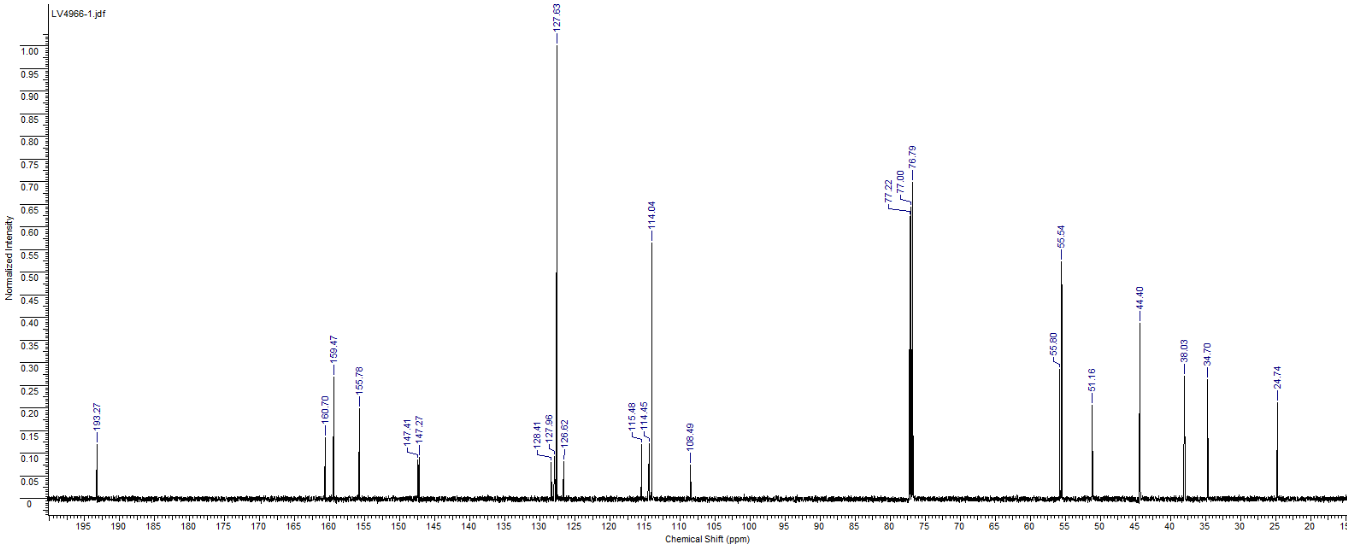

Figure S11. ^{13}C NMR spectrum of 1-{(4*E*)-8,9-dimethoxy-6-[1-(4-methoxyphenyl)-1*H*-tetrazol-5-yl]-3-methyl-1,2,3,6-tetrahydro-3-benzazocine-5-yl}-ethanone **2b.**

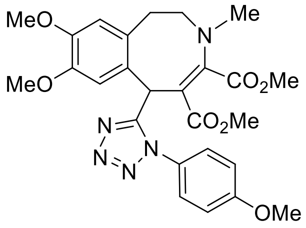

2c

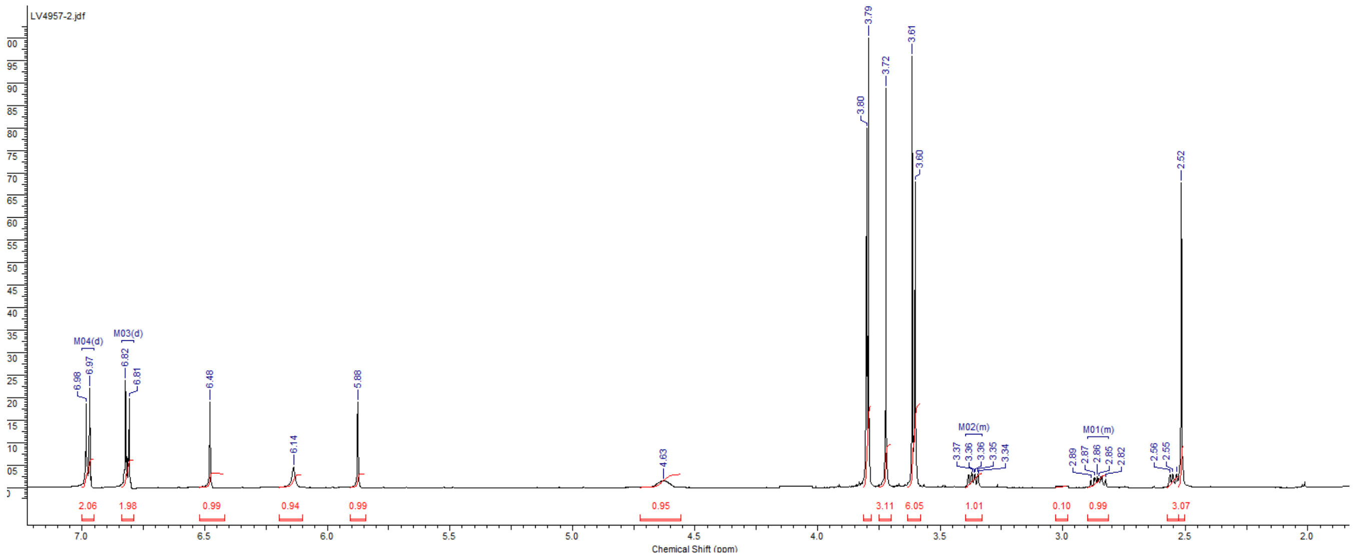

Figure S12. ^{1}H NMR spectrum of dimethyl (4*E*)-8,9-dimethoxy-6-[1-(4-methoxyphenyl)-1*H*-tetrazol-5-yl]-3-methyl-1,2,3,6-tetrahydro-3-benzazocin-4,5-dicarboxylate **2c.**

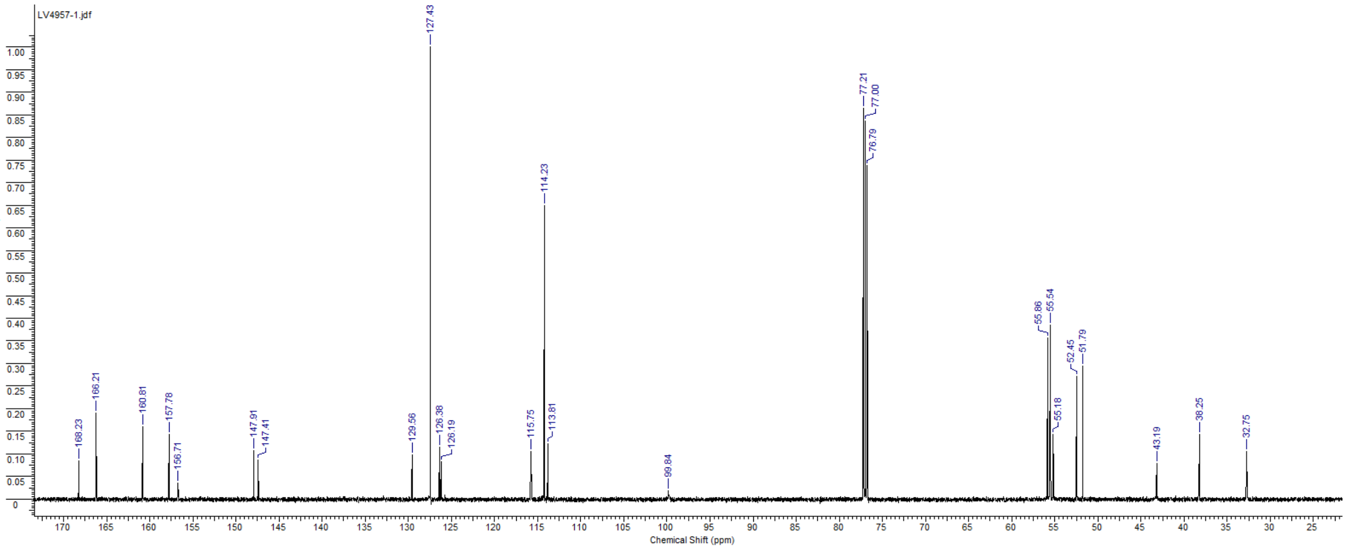

Figure S13. ^{13}C NMR spectrum of dimethyl (4*E*)-8,9-dimethoxy-6-[1-(4-methoxyphenyl)-1*H*-tetrazol-5-yl]-3-methyl-1,2,3,6-tetrahydro-3-benzazocin-4,5-dicarboxylate **2c.**

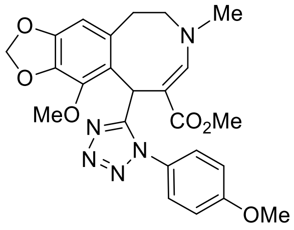

2d

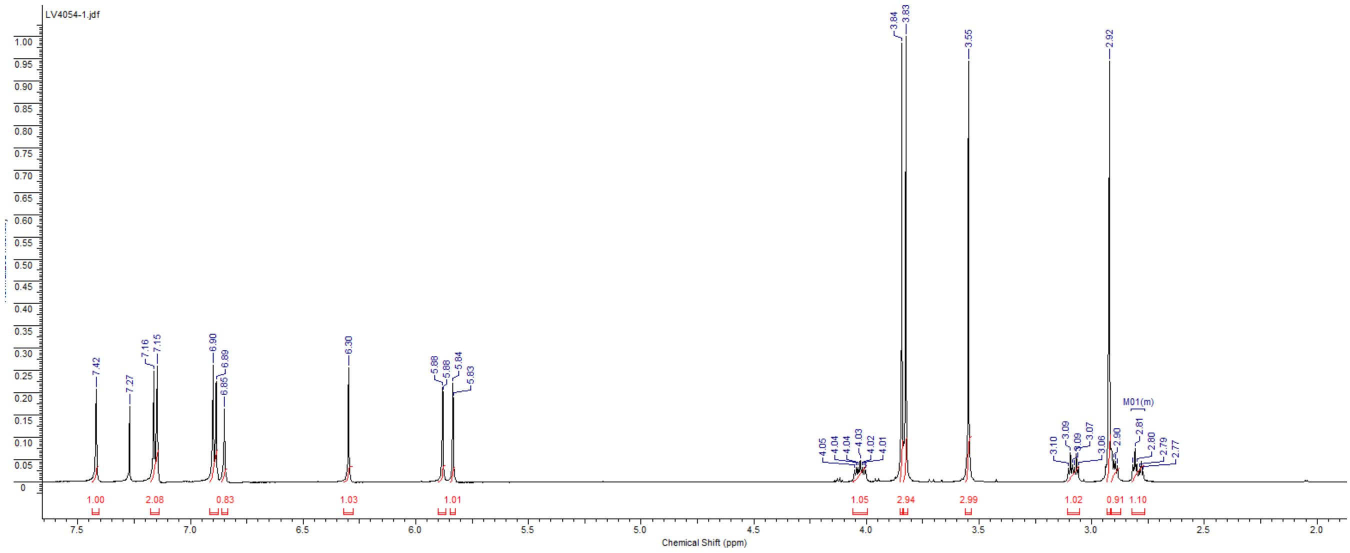

Figure S14. ^{1}H NMR spectrum of methyl (8*E*)-10-[1-(4-methoxyphenyl)-1*H*-tetrazol-5-yl]-7,11-dimethyl-5,6,7,10-tetrahydro[1,3]dioxolo[4,5-*i*][3]benzazocine-9-carboxylate **2d.**

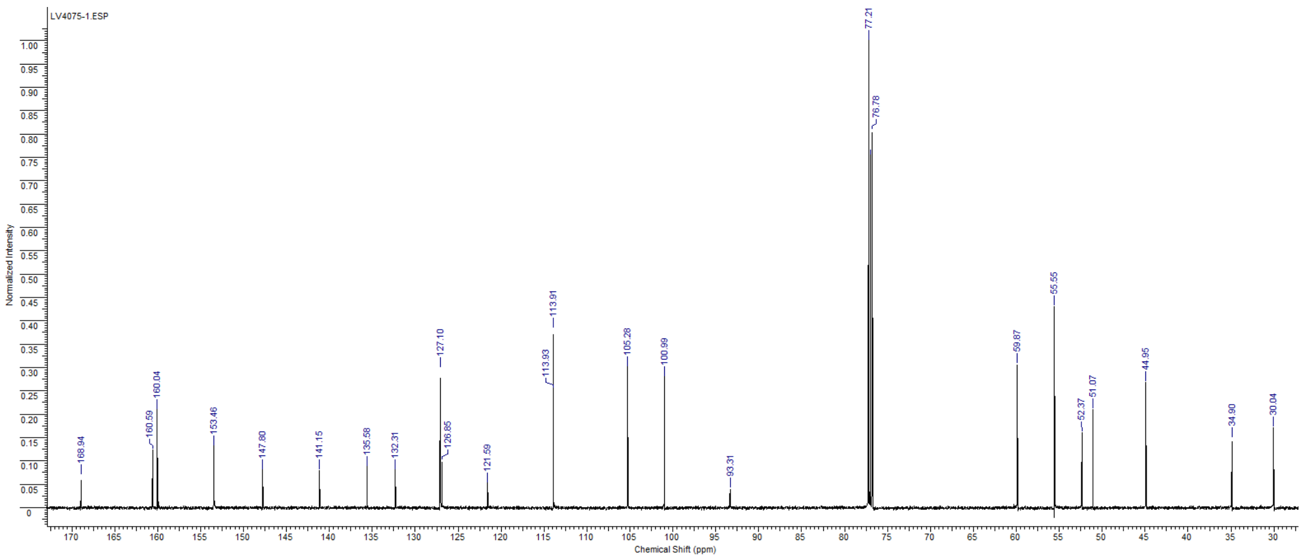

Figure S15. ^{13}C NMR spectrum of methyl (8*E*)-10-[1-(4-methoxyphenyl)-1*H*-tetrazol-5-yl]-7,11-dimethyl-5,6,7,10-tetrahydro[1,3]dioxolo[4,5-*i*][3]benzazocine-9-carboxylate **2d.**

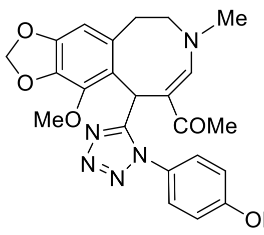

2e

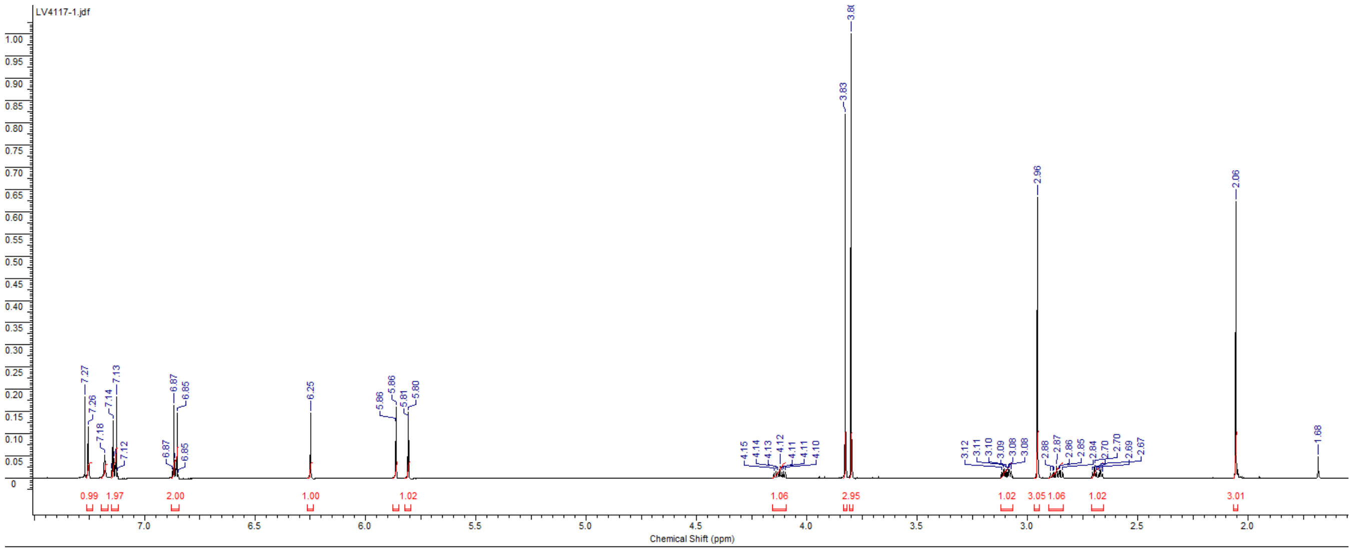

Figure S16. ^{1}H NMR spectrum of 1-{(8*E*)-11-methoxy-10-[1-(4-methoxyphenyl)-1*H*-tetrazol-5-yl]-7-methyl-5,6,7,10-tetrahydro[1,3]dioxolo[4,5-*i*][3]benzazocin-9-yl}ethanone **2e.**

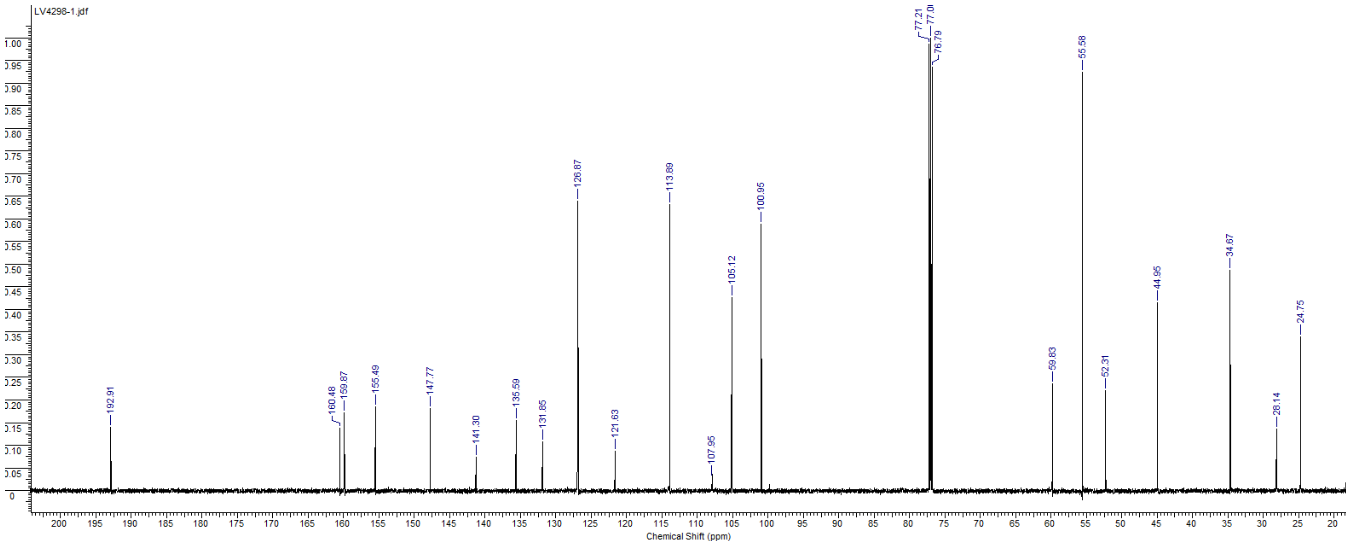

Figure S17. ^{13}C NMR spectrum of 1-{(8*E*)-11-methoxy-10-[1-(4-methoxyphenyl)-1*H*-tetrazol-5-yl]-7-methyl-5,6,7,10-tetrahydro[1,3]dioxolo[4,5-*i*][3]benzazocin-9-yl}ethanone **2e.**

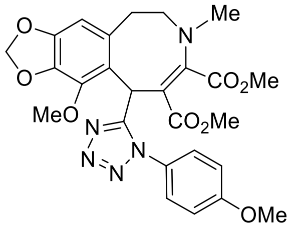

2f

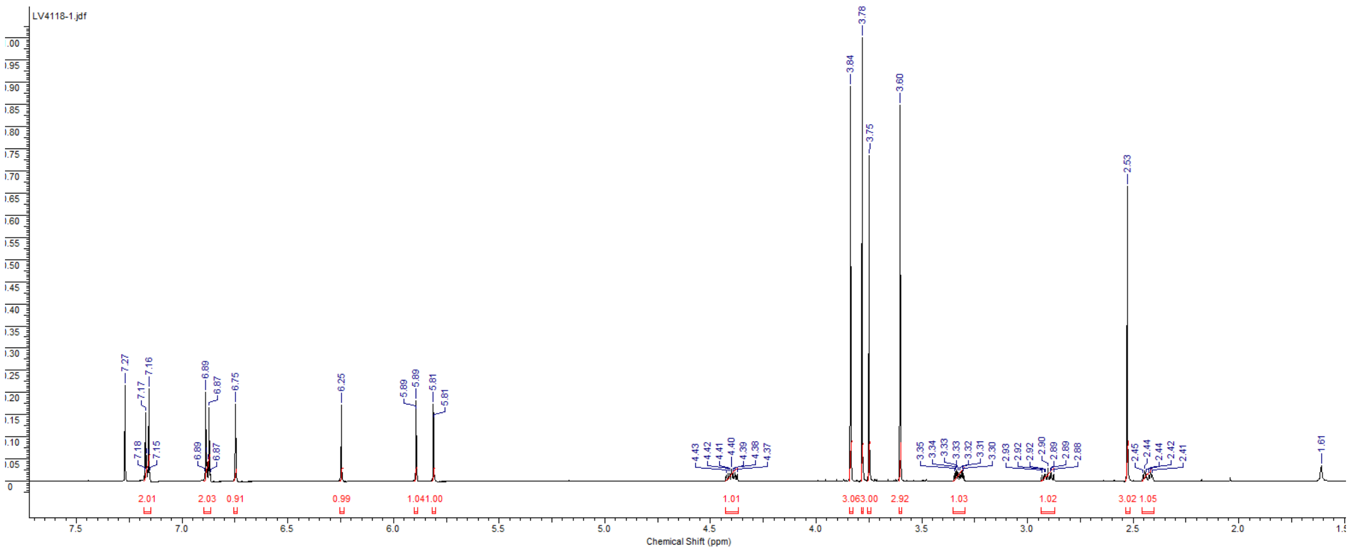

Figure S18. ^{1}H NMR spectrum of dimethyl (8*E*)-11-methoxy-10-[1-(4-methoxyphenyl)-1*H*-tetrazol-5-yl]-7-methyl-5,6,7,10-tetrahydro[1,3]dioxolo[4,5-*i*][3]benzazocin-8,9- dicarboxylate **2f**.

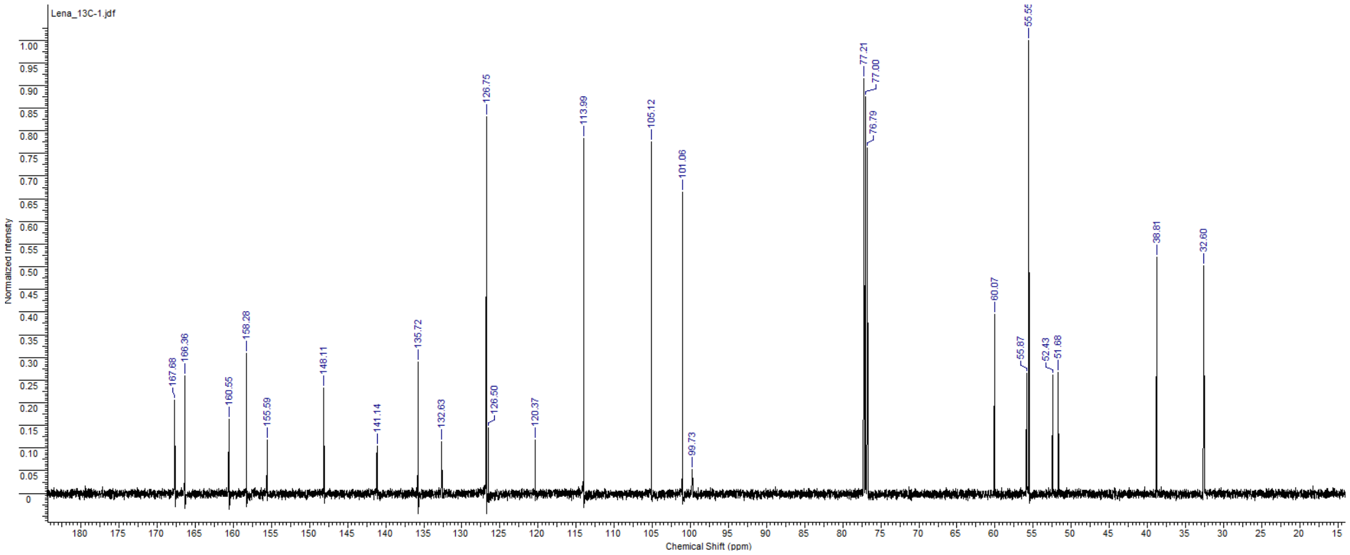

Figure S19. ^{13}C NMR spectrum of dimethyl (8*E*)-11-methoxy-10-[1-(4-methoxyphenyl)-1*H*-tetrazol-5-yl]-7-methyl-5,6,7,10-tetrahydro[1,3]dioxolo[4,5-*i*][3]benzazocin-8,9- dicarboxylate **2f.**

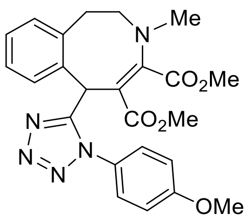

2g

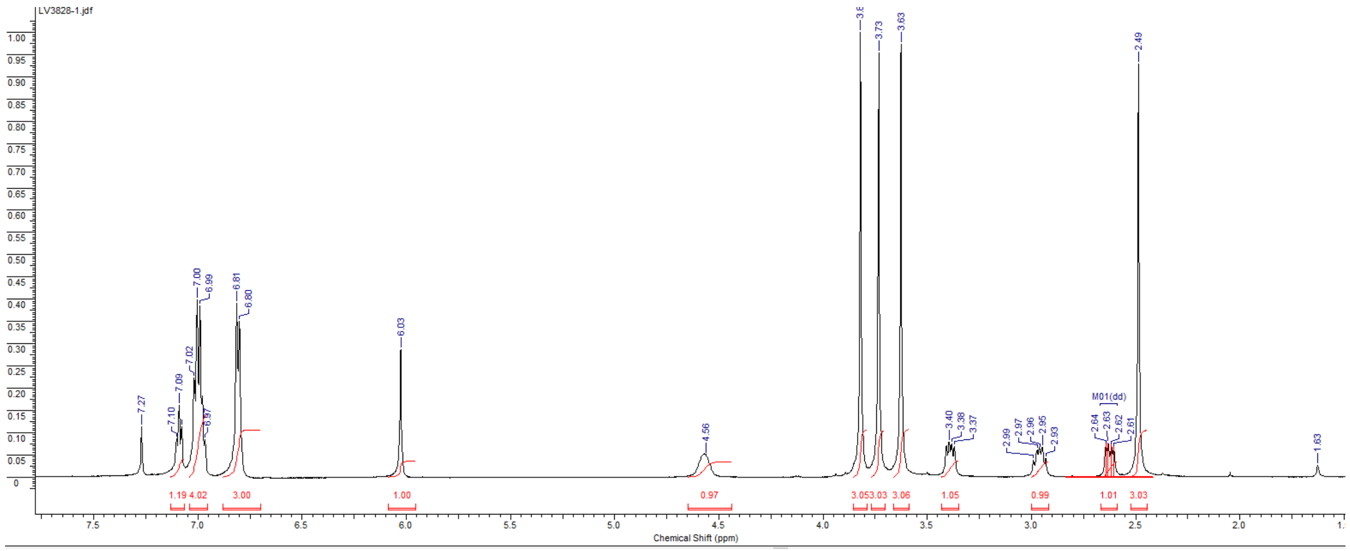

Figure S20. ^{1}H NMR spectrum of dimethyl 6-[1-(4-methoxyphenyl)-1*H*-tetrazol-5-yl]-3-methyl-1,2,3,6-tetrahydro-3-benzazocin-4,5-dicarboxylate **2g.**

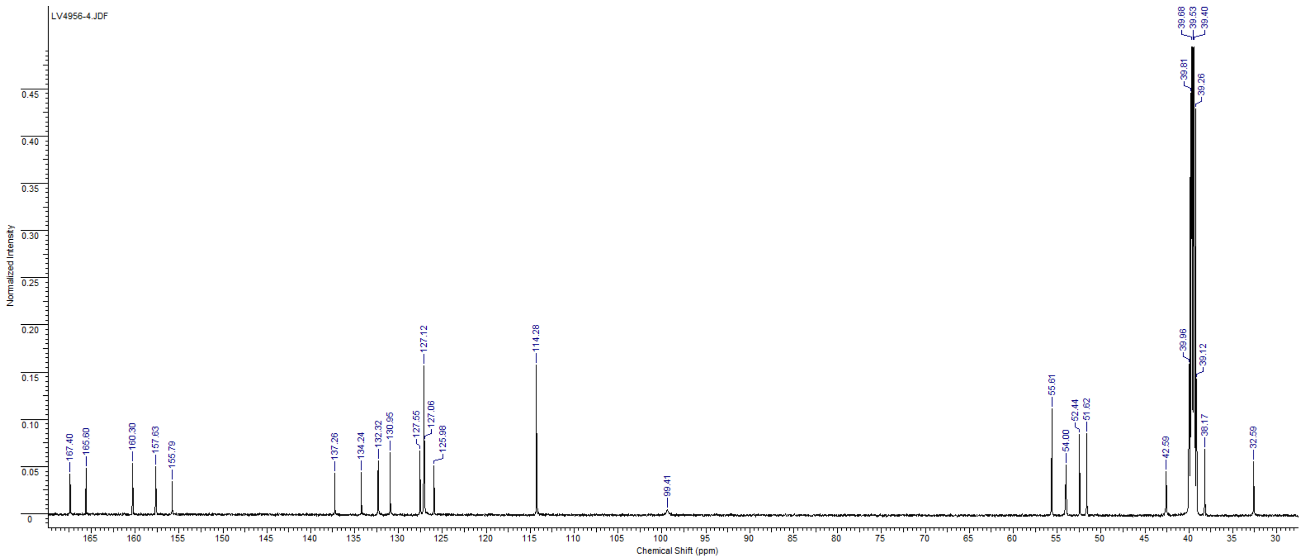

Figure S21. ^{13}C NMR spectrum of dimethyl 6-[1-(4-methoxyphenyl)-1*H*-tetrazol-5-yl]-3-methyl-1,2,3,6-tetrahydro-3-benzazocin-4,5-dicarboxylate **2g.**

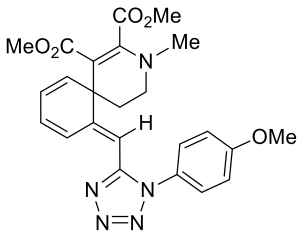

3

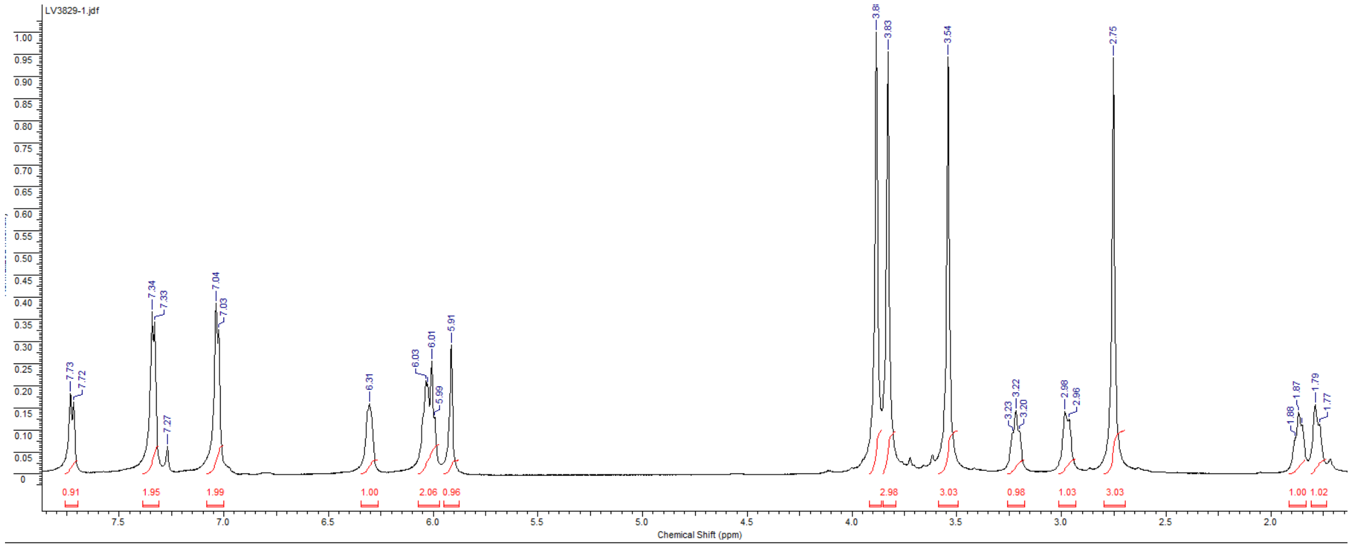

Figure S22. ^{1}H NMR spectrum of dimethyl (11*E*)-11-{[1-(4-methoxyphenyl)-1*H*-tetrazol-5-yl]methylidene}-3-methyl-3-azaspiro[5.5]undeca-1,7,9-triene-1,2-dicarboxylate **3.**

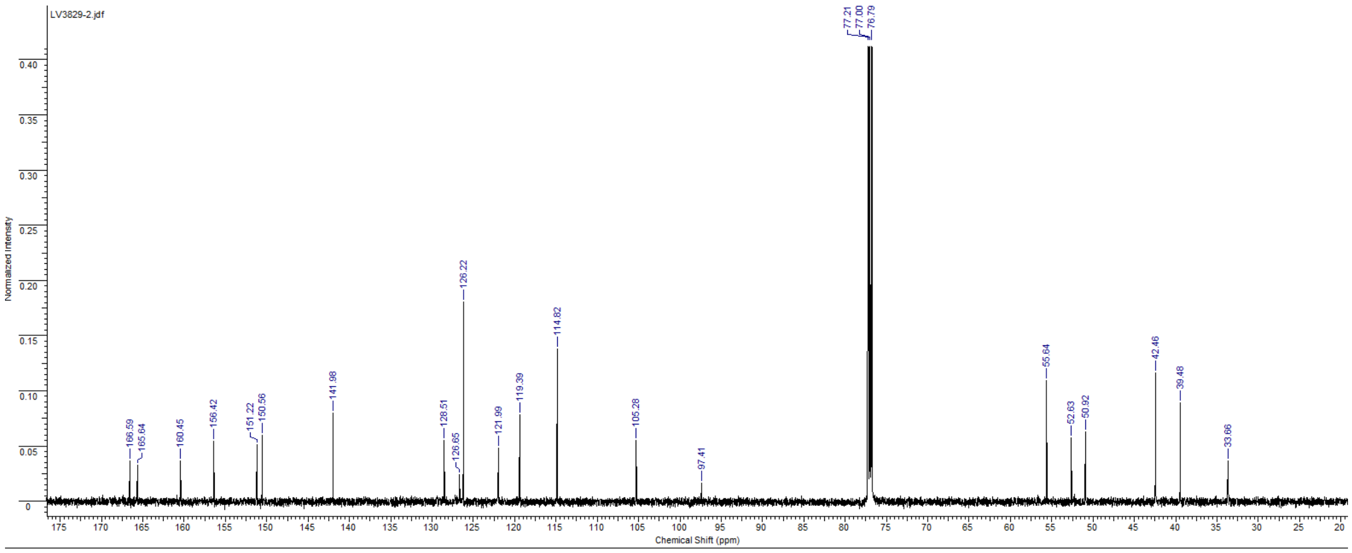

Figure S23. ^{13}C NMR spectrum of dimethyl (11*E*)-11-{[1-(4-methoxyphenyl)-1*H*-tetrazol-5-yl]methylidene}-3-methyl-3-azaspiro[5.5]undeca-1,7,9-triene-1,2-dicarboxylate **3.**

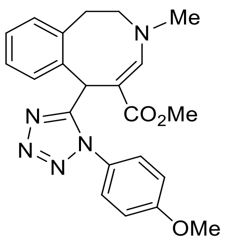

2h

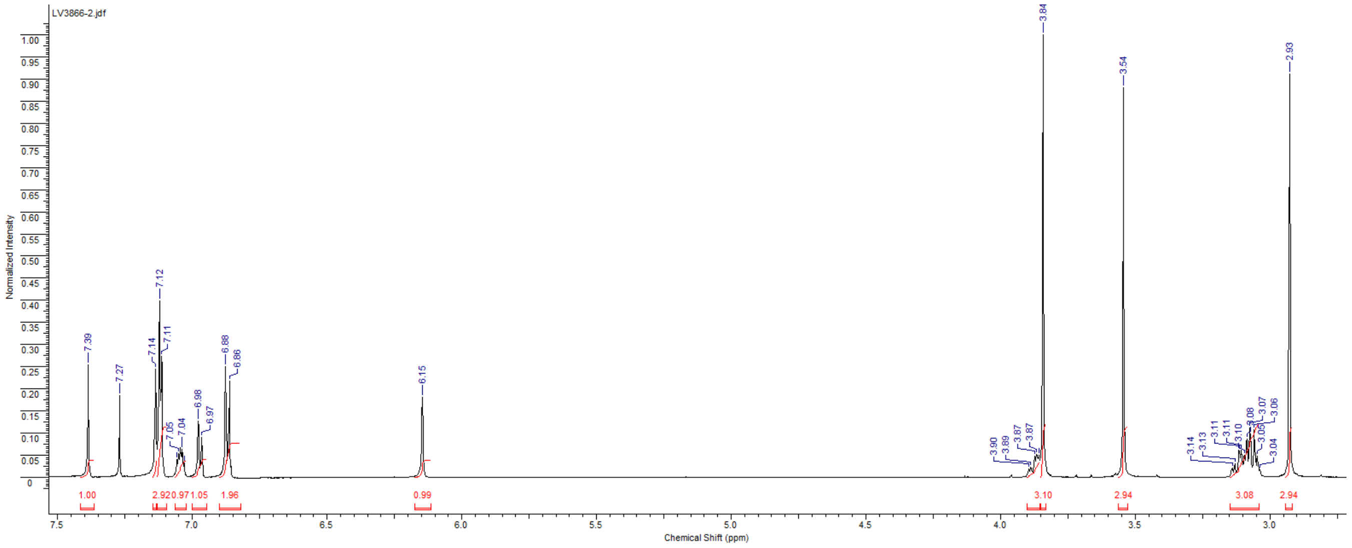

Figure S24. ^{1}H NMR spectrum of methyl (4*E*)-6-[1-(4-methoxyphenyl) -1*H*-tetrazol-5-yl]-3-methyl-1,2,3,6-tetrahydro-3-benzazocin-5-carboxylate **2h.**

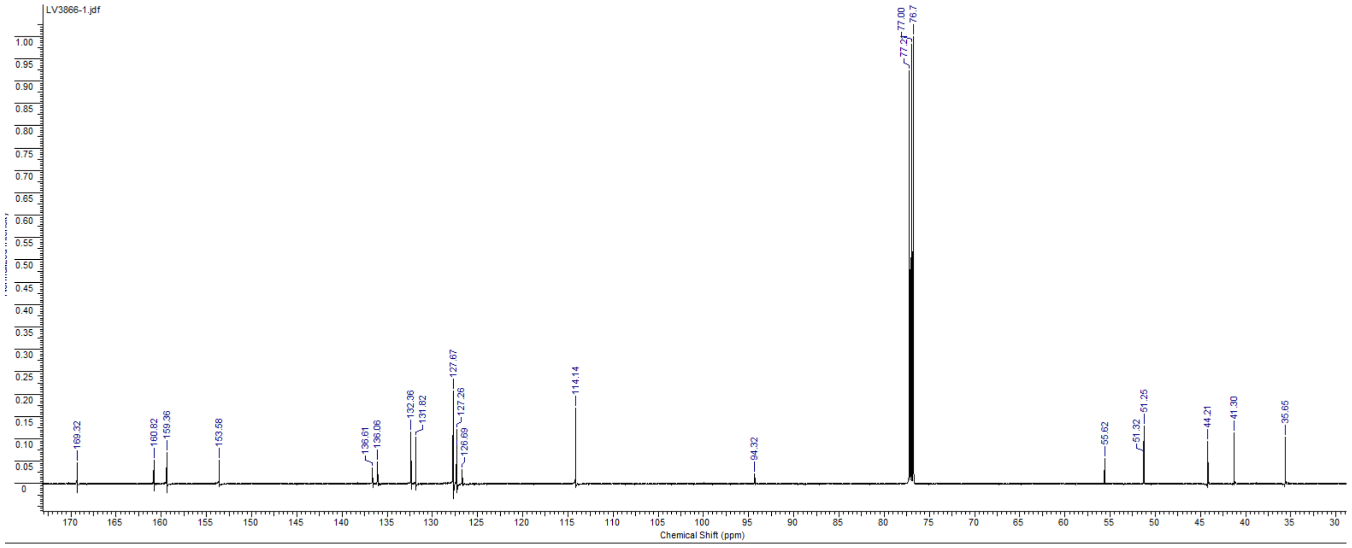

Figure S25. ^{13}C NMR spectrum of methyl (4*E*)-6-[1-(4-methoxyphenyl) -1*H*-tetrazol-5-yl]-3-methyl-1,2,3,6-tetrahydro-3-benzazocin-5-carboxylate **2h.**

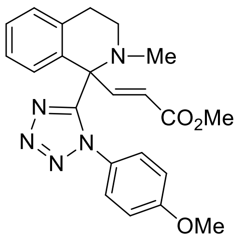

4a

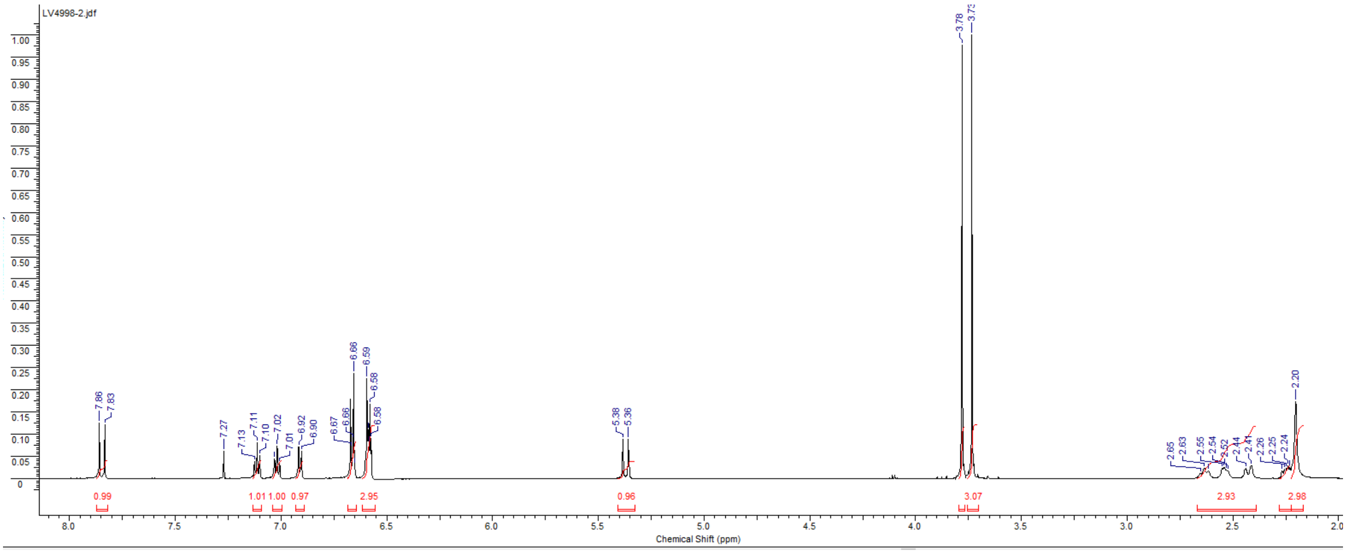

Figure S26. ^{1}H NMR spectrum of methyl (2*E*)-3-{1-[1-(4-methoxyphenyl)-1*H*-tetrazol-5-yl]-2-methyl-1,2,3,4-tetrahydroisoquinolin-1-yl}prop-2-enoate **4a.**

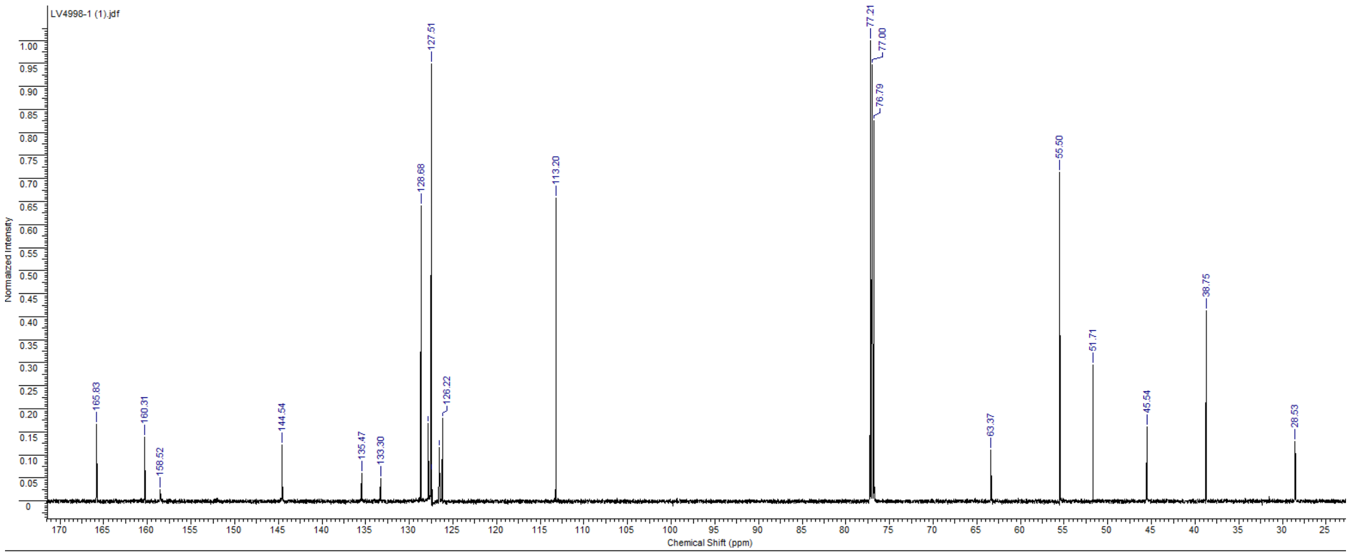

Figure S27. ^{13}C NMR spectrum of methyl (2*E*)-3-{1-[1-(4-methoxyphenyl)-1*H*-tetrazol-5-yl]-2-methyl-1,2,3,4-tetrahydroisoquinolin-1-yl}prop-2-enoate **4a.**

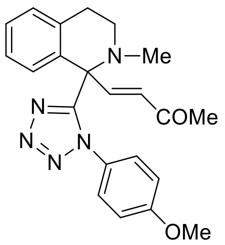

4b

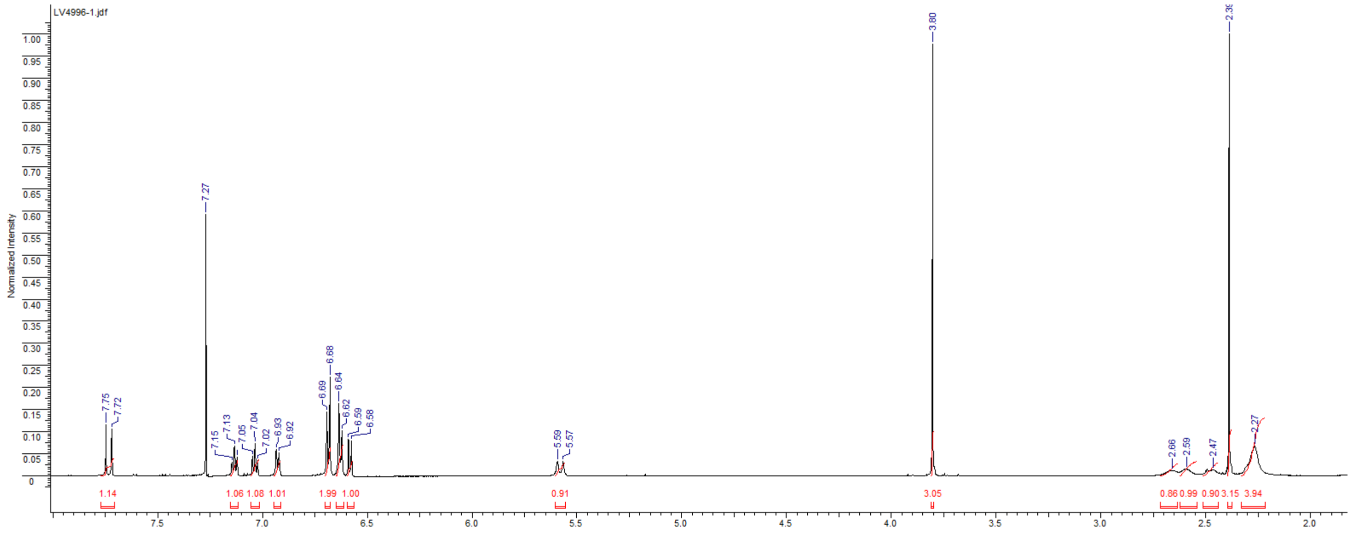

Figure S28. ^{1}H NMR spectrum of (3*E*)-4-{1-[1-(4-methoxyphenyl)-1*H*-tetrazol-5-yl]-2-methyl-1,2,3,4-tetrahydroisoquinolin-1-yl}but-3-ene -2-one **4b.**

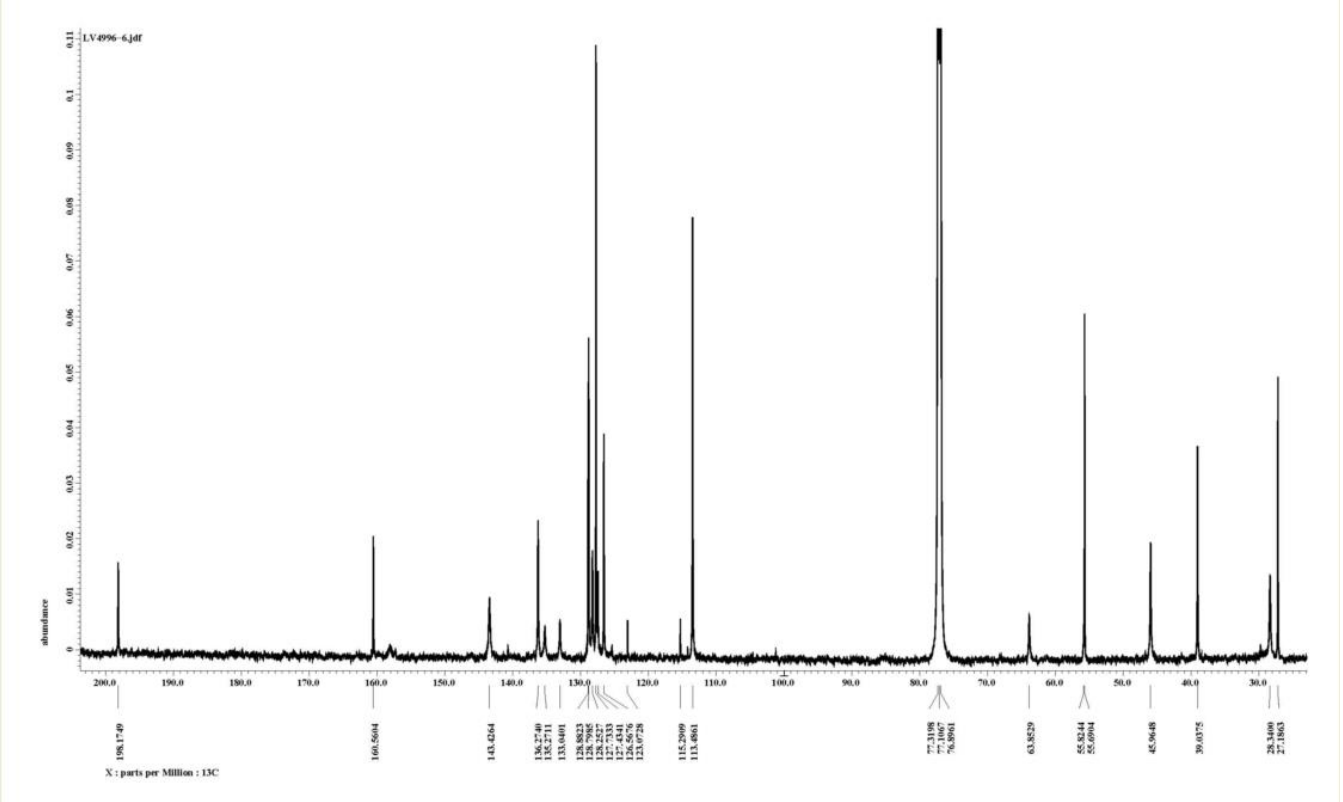

Figure S29. ^{13}C NMR spectrum of (3*E*)-4-{1-[1-(4-methoxyphenyl)-1*H*-tetrazol-5-yl]-2-methyl-1,2,3,4-tetrahydroisoquinolin-1-yl}but-3-ene -2-one **4b.**
